# Carbon Dot Nanoparticles Synthesized from Horticultural Extracts for Postharvest Shelf-Life Extension of Fruits and Vegetables

**DOI:** 10.3390/plants14162523

**Published:** 2025-08-13

**Authors:** Tshiamo B. Leta, Jerry O. Adeyemi, Olaniyi A. Fawole

**Affiliations:** 1 Postharvest and Agroprocessing Research Centre, Department of Botany and Plant Biotechnology, University of Johannesburg, P.O. Box 524, Auckland Park, Johannesburg 2006, South Africa; letatshiamo@gmail.com (T.B.L.); oadeyemi@uj.ac.za (J.O.A.); 2 South African Research Chairs Initiative in Sustainable Preservation and Agroprocessing Research, Department of Botany and Plant Biotechnology, University of Johannesburg, Johannesburg 2006, South Africa

**Keywords:** antioxidants, circular economy, food packaging, food preservation, food safety

## Abstract

The increasing demand for sustainable food preservation technologies has spurred interest in green-synthesized carbon dots (CDs) derived from horticultural produce, positioning them as a promising nanomaterial for prolonging the shelf life of perishable food products. Most of these green approaches offer renewable, low-cost nanoparticles with excellent ultraviolet (UV) light barrier capabilities, antioxidant, and antimicrobial properties. These features help protect food products from the growth of foodborne pathogens and retard oxidative spoilage to extend their shelf life through edible coatings and packaging. To this end, this review critically explores current breakthroughs in biosynthesis, characterization, and application of CDs generated from different agricultural extracts, the mechanism of action, and possible synergistic effects when paired with other food preservation agents, aligning with circular economic principles. Scalability challenges, regulatory limitations, and potential future directions are all explored to present a comprehensive understanding of the topic, paving the way for innovative preservation methods in the food industry.

## 1. Introduction

Despite the concerted efforts of the United Nations and various global bodies, there is a growing concern regarding food security due to multiple factors, including climate change, population growth, and food wastage. According to estimates from the World Factbook, the global population has been expanding at a rate of 1.06% per year since 2022, which was projected to be about 7.9 billion [1] and is estimated to exceed 9.73 billion in 2064 [2], highlighting the need for increased food production. In addition, food wastage resulting from factors such as foodborne pathogens during postharvest handling continues to contribute significantly to food shortages [3]. This issue is particularly pertinent for horticultural products such as fruits and vegetables, which have a high perishability rate. As a result, the infestation of these crops by different foodborne pathogens results in significant losses in quantity and quality of these products, rendering them less valuable or unsuitable for the market and leading to food waste [4]. According to the Food Waste Index Report 2024, 1.05 billion tons of food is projected to be wasted at households, food services, and retail, with an estimated cost of $940 billion (USD) annually [5]. Research has suggested that postharvest food losses in sub-Saharan Africa (SSA) are estimated to be worth over US$4 billion annually, sufficient to feed at least 48 million people [6]. To promote sustainable development, addressing this issue and transforming food waste into valuable products is imperative, as it is also a major source of environmental pollution [7,8]. Therefore, reducing food waste is an imperative global initiative that aligns with Sustainable Development Goal 12.3, which aims for a 50% reduction in food waste by 2050 [8,9].

Horticultural extract wastes are generated in significant quantities during food preparation and processing. Despite their low commercial value, these waste materials are highly abundant, inexpensive, and readily available, making them a valuable resource in material chemistry. Due to the presence of various bioactive compounds, these agricultural wastes can be converted into value-added products [10]. Through carbonization, the resulting agricultural waste extracts produce hydrophilic functional groups, such as carbonyl (C=O) and hydroxyl (-OH), along with a carbon dot core [11,12]. These functional groups, acting as effective chelating ligands, have numerous applications in the biomedical field, enhancing optical and physicochemical properties [10]. Therefore, one notable material that has garnered attention in recent years due to its accessibility of raw materials, ease of preparation, and useful biological applications is carbon dots (CDs). Carbon dots (CDs) were first accidentally discovered by researcher Xu and his team in 2004, at the U.S. Department of Energy’s Pacific Northwest National Laboratory during the purification of single-walled carbon nanotubes [13]. During this process, they found nanoparticles that emitted fluorescence, later identified as carbon dots. Due to the low toxicity and photoluminescence, this newfound material sparked significant interest and thus expanded its studies towards various studies focusing on their optical properties, biocompatibility, and environmentally friendly production methods, especially from natural and waste materials, such as plant extracts. This has positioned carbon dots as promising candidates for applications in biomedical imaging, sensors, biotechnology, drug delivery, and, more recently, food packaging and preservation.

Carbon dots are nanomaterials ranging from 1 to 10 nm in diameter and can be referred to as carbogenic quantum dots or carbon nanodots, depending on their structural properties, such as crystallinity and optical properties [14,15]. They can have an amorphous, graphite, C_3_N_4_ crystalline structure and, in most circumstances, sp^2^ hybridization followed by sp^3^ hybridization [16,17,18,19]. In contrast to semiconductor-based quantum dots, CDs present various advantages, including low cost, low toxicity, and strong chemical and optical inertness [20]. Carbon dots (CDs) possess distinct optical properties, such as high tunable photoluminescence (PL), excellent photostability, electrochemiluminescence, chemiluminescence, biocompatibility, simple surface functionalization, and a remarkable multi-photon excitation (up-conversion) property, partially due to their quantum confinement [20,21,22]. These properties enable their application in various environmental, energy, and healthcare sectors [22]. Recently, CDs have been proposed as a safe, economical, environmentally friendly, and sustainable functional material suitable for application in active food packaging [23]. Carbon dots have emerged as functional nanofillers for active food packaging materials owing to their beneficial characteristics such as excellent biocompatibility, high water solubility, chemical inertness, low cytotoxicity, and simple production method [23]. Furthermore, these nanoparticles exhibit outstanding properties, such as UV barrier, antioxidant, antifungal, and antibacterial activities [24,25].

Therefore, the increasing utilization of carbon dot nanoparticles in diverse fields, owing to their significant properties in food packaging, has necessitated this study. Consequently, this review investigates the various synthesis methods and biological activities of these carbon dot nanoparticles, rendering them suitable for formulating active food packaging materials for postharvest applications. Additionally, novel research avenues, experimental parameters and data, cytotoxicity, regulatory concerns, as well as advantages and limitations associated with the commercialization of carbon dot nanoparticles in biological and postharvest applications, with a specific emphasis on their implementation in the food industry as packaging materials, were also examined.

## 2. Overview of Physicochemical Properties and Various Approaches to Synthesizing Carbon Dots from Agricultural Extracts

The utilization of agro-waste, especially that of plant origin, which typically lacks monetary value, has been found in recent years to present a significant opportunity for preparing high-value nanoparticles with substantial applications in nanotechnology. The production of carbon dots by traditional chemical procedures involves the application of toxic chemicals, which pose a threat to the biological environment [18]. While initial pyrolysis setups are energy intensive [26], using free agro-waste offsets costs (for example, *Camellia sinensis* tea residue CDs [27]), enabling price competitiveness [28]. These initiatives align with circular economy models [29], resulting in sustainable investments from governments and investors receiving grants, subsidies, and tax breaks for creating and promoting the development of green innovative projects [30]. The development of sustainable nanomaterials using plant waste for environmentally sustainable packaging enhances brand image through customer interactions (green marketing, ESG reporting), therefore boosting consumer loyalty [31,32]. Therefore, green synthesis, which exploits plant-based materials as the source to synthesize CDs, has attracted massive attention in recent years [22]. This approach minimizes waste and contamination, making it environmentally friendly and sustainable [22]. Typically, agro-extracts are rich in carbon content, rendering them suitable as precursors for the synthesis of carbon dots (CDs). These materials offer numerous advantages in terms of safety, biocompatibility, and the capacity to meet the required synthesis conditions [33]. Moreover, various carbonaceous materials, including citric acid, Phenylenediamine, glucose, graphite, graphene oxide (GO), carbon nanotubes (CNTs), coal, petroleum coke, and plant extracts from leaves, stems, flowers, fruits, and vegetables, have been utilized to synthesize CDs, highlighting their biocompatible characteristics [34,35]. Carbon (C), hydrogen (H), nitrogen (N), sulfur (S) and oxygen (O) are abundantly present in natural resources in the form of carbohydrates, amino acids, and proteins and can also be used to dope carbon dots, thereby enhancing their physicochemical properties, such as size, morphology, and diverse fluorescence characteristics [35,36].

**Figure 1:**
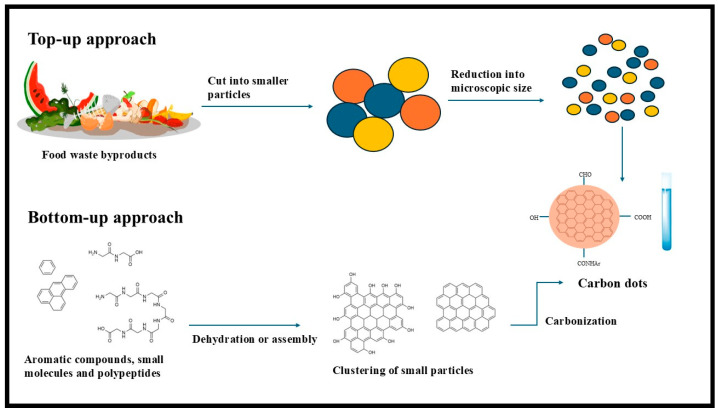
Schematic diagram representing top-down and bottom-up methods of obtaining carbon dots from diverse biological resources. Adopted and modified from [33] with permission from Elsevier.

There are currently two synthetic approaches to synthesizing CDs, which can be divided into two categories: “bottom-up” methods and “top-down” methods [37,38]. Carbon dots can be synthesized from diverse biological resources through top-down synthetic methods from waste and by-products, as shown in Figure 1. The top-down approach involves converting large sp^2^ carbon domains into small CDs using physical or chemical techniques, primarily chemical oxidation, and electrochemical synthesis [39]. Although the procedure is rapid, straightforward, and effective, the biogenic precursor only works with materials having large sp^2^ carbon domains, such as graphene or carbon fiber [40]. Nevertheless, the technique cannot regulate size or morphological distribution [39]. The bottom-up method generates CDs, CNTs, and polymer dots by dehydrating and further carbonizing small molecules with -C=O, -OH, -COOH, and -NH_2_ groups and polymers [33]. This process produces CDs with regulated size and shape by using small molecules as biogenic carbon source precursors and coupling organic molecules to create sp^2^ carbon domains using hydrothermal, carbonization, microwave-assisted, simple heating, and pyrolysis [35,39]. This approach has several advantages, such as consistent size, adjustable morphology, environmental friendliness, abundant and sustainable raw material sources, and low cost, as well as being time consuming and complex [39].

### 2.1. Biogenic Synthesis of CDs Using the Hydrothermal Method

**Figure 2:**
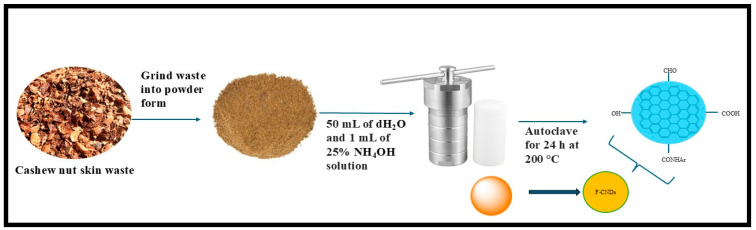
Hydrothermal synthesis of functionalized carbon nanodots from cashew nut (*Anacardium occidentale*) skin waste. Adopted and modified from [49].

This thermal-based method is one of the most widely employed techniques for nano-synthesis, including the production of carbon-based nanoparticles [41]. This method utilizes high-temperature aqueous solutions under elevated pressure to facilitate reactions that lead to the formation of materials at the nanoscale. It has gained prominence as a preferred means of synthesizing carbon dots, due to its straightforward preparation processes, considerable experimental requirements, and capacity to produce high quantum yield (QY%) for carbon dots [41]. This technique employs inexpensive instruments, such as an autoclave, to effectively synthesize carbon dots from biowaste materials [42]. The simplicity of synthesis, particularly when using agro-waste and other naturally occurring carbon sources, without the necessity for toxic chemicals, has attracted increased attention in the production of carbon-based nanoparticles [42]. Numerous studies have favored the hydrothermal approach because of its environmentally friendly, repeatable, non-toxic, low-cost, and operationally straightforward nature [43,44,45]. Consequently, these “bottom-up” approaches require an extensive amount of time and careful consideration of temperature control, hydrothermal treatment, and precursor selection [20]. As a result, many literature reports have documented the use of fruit waste extracts as carbon sources for carbon dots, including watermelon (*Citrullus lanatus*) peels [45], mango (*Mangifera indica*) peels [46], orange (*Citrus sinensis*) and lemon (*Citrus limon*) peels [47], Sapodilla (*Manilkara zapota*) fruits [48], and cashew nut (*Anacardium occidentale*) skin waste [49] using hydrothermal technique [33], as shown in Figure 2. Furthermore, the extent of carbonization is contingent upon the nature and quantity of the raw material employed [50]. Consequently, evaluating the raw material content for carbon dot synthesis is crucial for the selection of precursors, and the duration required to complete the carbonization reaction is indicated by the color changes of the resulting solution [50]. The solutions of carbon dots are generally orange, yellow, or brownish; if no color change is observed, this suggests that the duration has been insufficient for carbonization to occur [50]. Conversely, if the solution turns black, the synthesis duration has been excessively prolonged, resulting in carbon nanoparticles that lack luminescence [50].

The ease of synthesis and monitoring, alongside the use of plant extract, has thus led to increased interest in this approach as a route to CDs production. For instance, carbon dots have been synthesized from grapefruit peel using the hydrothermal technique. Briefly, the grapefruit (*Citrus × paradisi*) peel was initially sliced and cleaned with ultrapure water and dried at 60 °C [51]. After mixing 1.0 g of the peel with 50 mL of ultrapure water, the resulting mixture was filtered using cotton and Whatman No. 40 filter paper. A 50 mL Teflon-lined autoclave was then filled with 25 mL of the filtrate, and it was heated to 190 °C for 12 h. The product was centrifuged at 15,000 g for 15 min, placed in an MW 1000 dialysis bag to perform dialysis, and then freeze-dried [51]. These materials exhibited a size of 4.2 nm and a spherical morphology, which were subsequently utilized in photoluminescence immunoassays to detect p53 [52]. In addition, carbon dots doped with nitrogen (N-CDs) were synthesized from dwarf banana (*Musa* spp.) peel at 200 °C over 24 h; the resulting solution was cooled and passed through a 0.22 μm-pore-size mixed cellulose membrane to remove aggregated particles [52]. The final product was spherical particles with a diameter of 4.0 nm and with a quantum yield of 23% [53]. However, it should be noted that the hydrothermal process for producing bifunctional carbon dots necessitates a substantial amount of energy and the use of costly apparatus [54]. Moreover, this method may compromise sensitive chemicals present in the raw materials, potentially diminishing the antioxidative efficacy of the resultant carbon dots [55]. The application of hydrothermal conditions improves the reagents’ chemical and physical interactions, increases their solubility, and facilitates the formation of carbonaceous structures [41]. Table 1 summarizes other reports on the application of the hydrothermal method for synthesizing CDs using agricultural extracts.

### 2.2. Biogenic Synthesis of CDs Using the Microwave-Assisted Method

**Figure 3:**
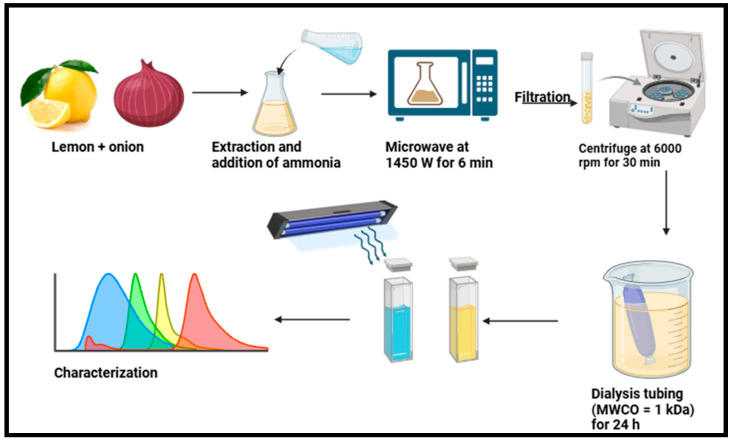
Schematic diagram representing the synthesis of CDs using lemon (*Citrus limon*) and onion (*Allium cepa*) via a microwave-assisted method. Adopted and modified from [65].

The application of microwaves for producing nanomaterials is also becoming increasingly prevalent and is regarded as an excellent approach to synthesizing CDs because it supplies the precursor solution with effective and consistent energy, facilitating the synthesis of CDs [41]. Microwave radiation addresses limitations with the traditional heating methods used to synthesize nanomaterials, such as the propensity of insoluble chemicals to cause heterogeneous heating, which increases the size of nanomaterials [84]. The microwave-assisted method provides a safe, affordable, and useful heating method owing to its significant energy efficiency, which increases the yields of the intended products [54,85]. The microwave-assisted approach may quickly heat materials with a high dielectric constant, and this approach can readily penetrate polymers and ceramics and be reflected by metal [86]. In addition to requiring less time, instantaneous microwave heating of the medium allows for real-time temperature adjustment by varying the power, possibly conserving a significant amount of energy [44]. Furthermore, this technique permits the manufacture of CDs from carbon precursors under the homogeneous heating effects of utilizing a domestic microwave oven [65,87,88]. However, there is no uniformity in the distribution of size [41]. Compared to the hydrothermal approach, the microwave-assisted method can heat the target molecules directly rather than convectively or conductively [65]. This technique also considers two parameters, namely the power output and time [51]. The microwave approach is more popular than the others because of its quick reaction time, low energy usage, simplicity of use, and environmental friendliness [89]. Nonetheless, for normal CD production, the intricate processes need to be made simpler [54]. Monte-Filho et al. [65] synthesized CDs using lemon (*Citrus limon*) and onion (*Allium cepa*) via microwave, as shown in Figure 3. The CDs were synthesized using Quince (*Cydonia oblonga*) fruit as a carbon source through powder microwave irradiation at 850 W for 1 min. Moreover, the CDs were stimulated at 350 nm and exhibited a QY of 8.55% with peak emission intensity at 450 nm [75]. Orange peel and banana (*Musa* spp.) peel were used as carbon sources using the microwave-assisted method for 2–5 min [70]. These findings demonstrate the wavelength intensity of CDs banana (*Musa* spp.) peel at 501 nm and CDs orange (*Citrus sinensis*) peel at 498 nm [70]. Other reports on the application of the microwave-assisted approach for synthesizing CDs using agricultural extracts are summarized in Table 1.

### 2.3. Biogenic Synthesis of CDs Using Chemical Oxidation Approach

**Figure 4:**
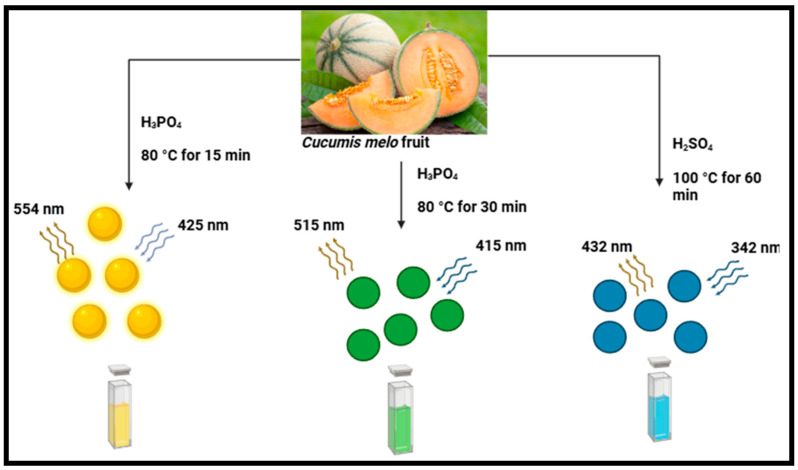
Synthesis of multi-emission CDs from Muskmelon (*Cucumis melo*) fruit via acid oxidation method. Adopted and modified from [69].

Chemical oxidation is a novel technique used to synthesize CDs at a commercial level. This approach involves the application of plant precursors and strong oxidants such as nitric acid (HNO_3_) and sulfuric acid (H_2_SO_4_) [44]. Acid oxidation has been applied to exfoliate and break down bulk carbon into nanoparticles while concurrently adding hydrophilic groups, such as carboxyl or hydroxyl groups, on the surface of the CDs [16]. Despite the impressive photoluminescence displayed by CDs produced through chemical oxidation, their chemical toxicity and environmental impact should not be disregarded 90]. Some of the oxidizing agents used could be burning or an explosion [90]. The most used top-down synthetic approach to synthesizing CDs is chemical oxidation due to its numerous outstanding benefits, notably easy size control, low cost, high yield, and high quality [91]. The disadvantage pertaining to chemical oxidation is that there is no uniformity in the distribution of size [41]. Numerous chemical agents have been employed, such as H_2_SO_4_ as a porosity controller and KOH as a high surface area promoter [92]. Furthermore, many impurities could occur if carbon dots are produced from biomass or waste materials rather than pure chemicals [92]. Several studies have employed this technique, such as those involving the use of pineapple (*Ananas comosus*) at 80–100 °C for 15–60 min to synthesize CDs through acid oxidation [42]. The QY of the produced CDs was determined to be 18.0%, 37.6%, and 44.7% for B-(blue), G-(green), and Y-(yellow) CDs, respectively. Quinine sulphate and rhodamine 6G were used as reference materials for the estimation [42]. Similarly, muskmelon (*Cucumis melo*) fruit was used to synthesize CDs using acid oxidation at 80 °C for 15–30 min [69], as shown in Figure 4. The prepared CDs demonstrated 14.3%, 26.9%, and 7.07% QY at stable emissions. Moreover, they had distinct emission wavelengths when stimulated at 342, 415, and 425 nm, respectively, at 432, 515, and 554 nm [69]. Other reports on the application of the chemical oxidation method to synthesize CDs using agricultural extracts have been presented in Table 1.

### 2.4. Biogenic Synthesis of CDs Using the Ultrasonic Method

**Figure 5:**
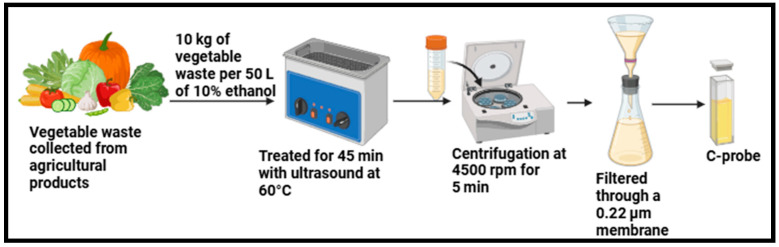
Schematic diagram representing the synthesis of C-probe carbon dots from vegetable waste using an ultrasound approach. Adopted and modified from [82].

The ultrasonic technique has been widely adopted for synthesizing CDs due to its facile, environmentally friendly approach, cost effectiveness, and ability to produce high-quality nanomaterials [93,94]. This approach has several elements, including surface morphology, size, chemical composition, solubility, and aggregation, which affect how CDs form or change during this process [95]. The CDs are usually less than 10 nm and have a constant size distribution due to the homogenous energy distribution throughout the solution [44]. In contrast to other synthesis processes, such as hydrothermal or chemical vapor deposition, the ultrasonic approach requires no harsh reaction conditions, such as high temperatures or pressures [54]. Ultrasonic treatment has a very high energy of ultrasonic sound waves and can break down large carbon materials [91]. The process of carbonization produces CDs with larger particle sizes and less homogeneous surface morphology; on the contrary, the ultrasonic approach can compensate for these drawbacks [96]. This approach produces large yields of nanoparticles with desirable surface functions. Consequently, ultrasonic synthesis continues to encounter challenges due to the thermal effect of ultrasonic waves, substantially less than that of direct heating or the microwave-assisted method [97], and requires a high energy cost [41]. Thus, there is a great deal of room for improvement in the ultrasonic approach to CD synthesis [44]. This process is renowned for its enhanced precursor reactivity and little external energy use [54]. Kim et al. synthesized CDs from vegetable waste/ethanol, which were treated at 60 °C for 45 min using an ultrasonic machine (40 kHz, Ultrasonics UC-05, Lab Companion, Daejeon, Republic of Korea), as shown in Figure 5 [82]. The process that is essential to the production of CDs is known as acoustic cavitation. Acoustic cavitation produces small bubbles in the solution, which expand and collapse rapidly, producing extremely elevated temperatures (up to 5000 K) and pressures (up to 1000 atm) for very short durations. Chemical bonds within the organic precursors can be broken by these extremely localized conditions, forming carbon atoms that nucleate and develop into carbon dots [93]. The solution was centrifuged at 4500 rpm for 5 min to purify an aqueous solution and remove the agglomerated particles [82]. To further remove large particles, the resultant solution containing the C-probes was filtered multiple times through a 0.22 μm membrane before being freeze-dried. Carbon dots synthesized derived from biowaste via the ultrasonic approach displayed high fluorescence, biocompatibility, water solubility, and chemical stability, rendering them highly versatile for environmental and biological applications [82]. Because of their fluorescent properties, CDs are utilized frequently in bioimaging, making them useful for the tracking of biological processes at the cellular level [98]. Their small size, non-toxic nature, and biocompatibility make them suitable for this application since they may be safely incorporated into biological systems [82]. To increase the CDs’ water solubility and suitability for biological applications, functional groups, such as hydroxyl, carboxyl, or amine groups, are added to their surface during production [44]. These functional groups also considerably increase the optical properties of the CDs, particularly their surface defect state fluorescence, which is a crucial aspect in applications, such as bioimaging, biosensing, and chemical sensing [51,98]. In contrast, the mechanism of fluorescence emergence is not clearly explained [54]. Nonetheless, CDs are being adopted more often in drug delivery systems owing to their functional surfaces, which enable therapeutic molecules to conjugate, enabling exact targeted drug delivery, particularly for cancer treatment [99,100]. Their fluorescence is quenched or amplified in the presence of analytes, which renders them suitable detectors for pollutants, heavy metals, and other toxic substances due to their sensitivity to a variety of chemical stimuli [51,54,69]. This versatility, combined with the environmentally friendly methods and scalability, guarantees its continued relevance in nanotechnology [44].

### 2.5. Biogenic Synthesis of CDs: Pyrolysis and Carbonization Method

Pyrolysis has become a powerful method to produce luminous CDs in recent years by employing small carbon structures as precursors [41]. Over the past 20 years, pyrolysis technology has advanced rapidly, developing several linked, microwave cracking, and rapid pyrolysis technologies [26,101]. Pyrolysis carbonization is a traditional method used to synthesize CDs and display low toxicity, simplicity, and convenience [102]. Moreover, the composition, yield, and quantum yield of CDs are influenced by pyrolysis parameters such as reactor system type, heating duration, temperature, pressure, and catalyst presence [101]. The basic procedures of heating, dehydrating, degrading, and carbonization are necessary at high temperatures to transform the molecules containing organic carbon into CDs [41]. Also, this process involves the cleavage of carbon initiators into carbon nanoparticles by high concentrations of alkaline [41]. However, the pyrolysis method often results in non-uniform particle size distribution and is energy intensive; the incorporation of simple modifications, such as sonochemical or ultrasonic treatments, may improve uniformity since they are easy to operate [41].

**Figure 6:**
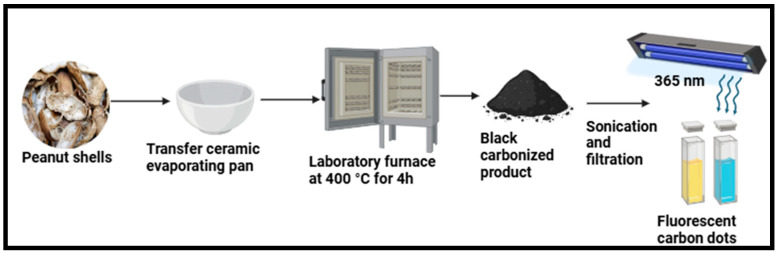
The synthesis of peanut (*Arachis hypogaea*) shells using the pyrolysis method. Adopted and modified from [73].

For instance, nitrogen-doped CDs produced using pyrolysis were dissolved in water and subjected to an ultrasonic reaction, which resulted in CDs that were spherical in shape with a particle size of 8 nm and QY of 14% [103]. Given its low QY and large equipment requirements, it is imperative to seek better solutions to keep up with future advancements [44]. Ma et al. synthesized CDs using peanut (*Arachis hypogaea*) shells as a carbon source via a one-pot pyrolysis approach optimized using central composite design, as illustrated in Figure 6. The optimum reaction conditions were 70 g, 4 h, and 400 °C using response surface methodology [73]. Moreover, the synthesized CDs had a QY of 10.58% [73]. Tan et al. used sago (*Metroxylon sagu*) industrial waste as a carbon source to produce CDs using a thermal pyrolysis method [77]. The powdered sago waste was weighed and then placed in a crucible within a laboratory furnace. After heating, the resulting sample was kept at the desired temperature for 1 h without any gas flow, with a temperature ranging from 250 to 450 °C. The final product was heated and allowed to settle at 25 °C in a desiccator before being combined to create a combination with a mass concentration of 10 g/L. Then, a volumetric flask containing 10 mg of heated sago waste was weighed and then mixed with 10 mL of ultrapure water. The resulting mixture was homogenized for 2 h using a Branson 5510 Ultrasonic Cleaner, and the slurry was centrifuged for twenty minutes at 13,400 rpm using an Eppendorf Minispin to eliminate the larger particles. Subsequently, the supernatant was collected and diluted 1:2 for optical examination. The carbon dots had an irregular shape and ranged from 6–17 nm; they displayed a strong fluorescence emission at 390 nm with an ideal excitation wavelength of 315 nm [77]. Gunjal et al. produce waste tea (*Camellia sinensis*) residue CDs from surplus and kitchen waste biomass by using a simple carbonization process, which is less expensive and more environmentally friendly than previous techniques [27]. Other reports on the application of hydrothermal, microwave-assisted, ultrasonic, chemical oxidation method, and pyrolysis carbonization to the synthesis of CDs using agricultural extracts have been presented in Table 1.

## 3. The Major Factor Affecting the Properties of CDs

There are various parameters that influence the physicochemical properties of CDs, including starting material, reaction temperature, pH, and type of solvent (polar, nonpolar, and protic), which can be optimized for higher yield [35,104]. The factors include quantum size effects, surface passivation, surface defect states, and bandgap transition [105,106]. Consequently, the variance in emission peaks is due to the varied sizes of CDs. The energy gap increases with decreasing CD size because of the quantum confinement effect, and larger particles are stimulated at longer wavelengths, whereas smaller particles are excited at shorter wavelengths [107]. Among these factors, one of the most significant for understanding the photoluminescence characteristics of CDs is the surface state [108]. The main contributory factor of surface states of CDs is surface oxidation, which can result in surface defects and change the photoluminescence of the synthesized CDs [109]. As a result, a redshift in the emission spectrum of CDs occurs because of the formation of additional surface defects to capture more excitons as the degree of surface oxidation increases [109]. Moreover, the fluorescence properties of CDs can be significantly impacted by functional groups on the surface of CDs. Nevertheless, despite the variations in their fundamental structures, carbon dots can be readily functionalized with a range of functional groups, such as amino, carbonyl, epoxy, hydroxyl, and carboxyl groups [110]. This section discusses the effects of some of the synthesis conditions on the features of CDs.

### 3.1. The Impact of Biological Carbon Precursor

There are different raw materials used to synthesize CDs that may have an impact on their fluorescence properties. Different precursors consist of various functional groups that can be applied to the surface of CDs throughout the manufacturing process [111]. For instance, a clear difference was apparent in some properties of CDs synthesized from pineapple and cucumber peels using the same synthetic approaches [112]. The CDs from pineapple and cucumber were spherical in morphology, with a size of ~50 nm. The X-ray diffraction (XRD) pattern of the CDs displayed an intense peak at 29.781° and 31.428° and 29.781°, respectively. The highest emission of CDs from cucumber (*Cucumis sativus*) peels was captured at 502 nm with a 440 nm excitation wavelength [112]. Whereas CDs from pineapples (*Ananas comosus*) recorded a maximum fluorescence emission at 487 nm with a 360 nm excitation wavelength. Boruah et al. synthesized CDs from sugarcane (*Saccharum officinarum*) bagasse (S-CDs), garlic (*Allium sativum*) peels (G-CDs), and taro (*Colocasia esculenta*) peels (T-CDs) using the ultrasonic-assisted wet-chemical-oxidation approach [78]. The diameter (nm) range of the synthesized CDs was 8–12 nm, 8–12 nm, and 8–11 nm for S-CDs, G-CDs, and T-CDs, respectively. These nanomaterials displayed different quantum yields of 4.5%, 13.8%, and 26.2%, respectively. Fruit peel waste from orange (*Citrus sinensis*) and lemon (*Citrus limon*) was also used to synthesize CDs via the carbonization method at 180 °C [48]. The as-prepared CDs were spherical with diameters of 6.5 and 4.5 nm, respectively. The quantum yields of the CDs from orange (*Citrus sinensis*) and lemon (*Citrus limon*) were determined to be 16.8% and 15.5%, respectively.

### 3.2. The Effect of Reaction Time

Both reaction temperature and reaction time can exert a comparable effect on the optical properties of CDs, demonstrating that both parameters are of equal significance. A long reaction time could lead to over-carbonization and destroy the CD surface structure [113]. In contrast, a short reaction time will result in CDs with a diminished fluorescence emission attributed to inadequate carbonization of the precursor material, resulting in reduced graphitic structure development [64]. Also, the reaction time has a temperature-dependent influence on the optical properties of CDs. For instance, Bhamore et al. synthesized multi-colored CDs using sapodilla (*Manilkara zapota*) fruits using phosphoric acids (H_2_PO_4_) at 80 °C for 30 min for green CDs (G-CDs) and 80 °C for 15 min for yellow CDs (Y-CDs) [49]. When excited at 420 and 440 nm, the fluorescence emission intensity of G-CDs and Y-CDs was observed at 515 and 563 nm. The quantum yields of G-CDs and Y-CDs were 7.9 and 5.2%. These nanomaterials had average sizes of 2.9 and 4.5 nm, respectively. According to Bandi et al., the photoluminescence efficiency of CDs synthesized from onion (*Allium cepa*) waste using a hydrothermal approach was significantly affected by the reaction time [114]. As the reaction time increased, the quantum yield of CDs first increased and then decreased as the reaction time was further prolonged. Ngu et al. demonstrated a similar relationship between the quantum yield and reaction time of CDs synthesized from waste rice husk using the thermal-assisted carbonization method [115]. The findings indicated that a sulphuric acid concentration of 12 mol/L, a reaction temperature of 120 °C, and a reaction duration of 30 min were optimal for synthesizing CDs. The samples displayed a peak emission at 439 nm, indicated by blue luminescence [115].

### 3.3. The Effect of Synthesis Temperature

**Figure 7:**
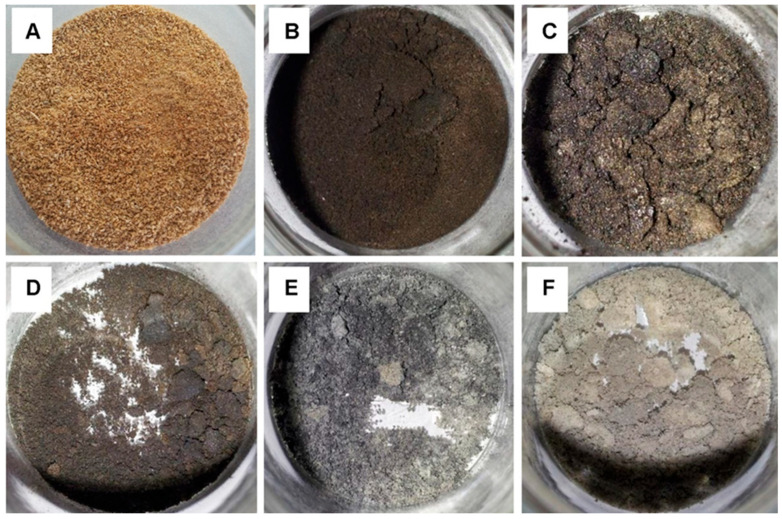
Visual images of sago wastes treated at various temperatures before pyrolysis (**A**), after pyrolysis at 250 °C (**B**), 300 °C (**C**), 350 °C (**D**), 400 °C (**E**), and 450 °C (**F**). Reproduced from [77] with permission from Elsevier.

Carbonization, an energy-absorbing (endothermic) process that is necessary to produce fluorescent CDs using biomass wastes as carbon sources, and temperature plays a crucial role in this process [109]. Tan et al. conducted an in-depth investigation on the impact of pyrolysis temperature on the properties of sago waste-derived CDs, and thermal degradation was applied to a sample of sago waste at different temperatures [77]. As shown in Figure 7, the initial color of the sago waste appeared to be golden brown before it was heated in a furnace. After heating to 250 °C, a final residue with a dark brown color was produced. The product of the sample treated at 300 °C was black in color and demonstrated improved sago waste carbonization. Samples that were heated to 350 °C and 400 °C produced a mixture of black and slightly ashy gray products. The sample heated at 450 °C produced an ashy product, indicating a complete decomposition of sago waste into dark residue. Additionally, the relationship between temperature and weight reduction was examined [77]. Sago waste treated at 250 °C exhibited the least amount of weight loss, with mass reduction (over 60%) that can be attributed to the substantial breakdown of the organic contents, indicating that the sago waste was severely decomposing into carbonaceous wastes. Weight loss at 350–450 °C was relatively unchanged, suggesting that the degree of weight loss was not significantly influenced by these temperature increases. It was determined that pyrolysis temperatures above 450 °C would not significantly impact weight loss because the majority of the waste’s constituents might have been drastically broken down into ashy residues [77]. Moreover, sage waste treated at 400 °C was the ideal temperature for carbonization and displayed the highest fluorescence emission intensity recorded at 390 nm with an optimal excitation wavelength of 315 nm. Sago waste treated at 250 °C demonstrated the lowest fluorescence emission intensity of 436 nm. A higher temperature can result in severe decomposition, whereas treatments below 400 °C may result in incomplete carbonization [77].

## 4. Characterization of Carbon Dots

**Figure 8:**
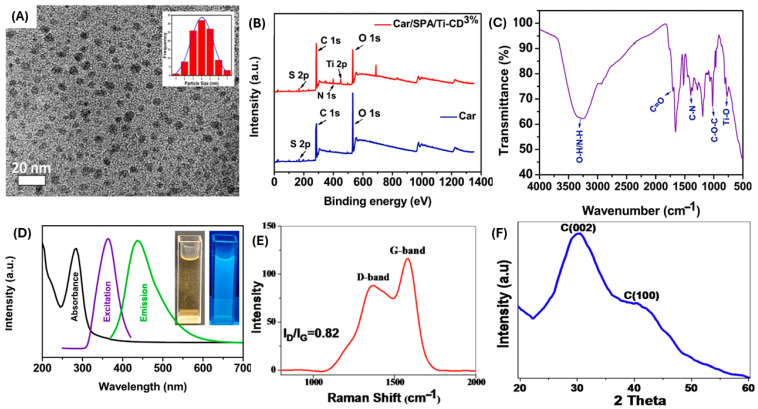
HR-TEM image of N-doped CDs (**A**), XPS spectra (**B**), FTIR spectrum of Ti-CDs (**C**), optical properties using UV–vis absorption (**D**), Raman spectrum of N-CDs (**E**), and XRD graph of N-doped CDs (**F**). Reproduced from (**A**,**E**,**F**) [120] and (**B**–**D**) [121] with permission from Elsevier.

The various synthesis techniques lead to a diversity of chemical structures for CDs. For instance, graphite quantum dots (GQDs) have one or multiple graphene layers, coupled with chemical groups along the edges, exhibiting anisotropy, where their lateral dimensions surpass their height [105]. Furthermore, GQDs have a unique crystallinity owing to the presence of a carbon core, which is equivalent to the (100) spacing between individual graphene dots on lacey support films [116,117]. Also, in comparison to carbon nanoparticles, which are spherical and without visible crystal lattices, carbon dots (CDs) have a visible crystal lattice [110]. The CDs typically have an interlayer distance of 0.34 nm or the (002) spacing of crystalline graphite CDs [110]. These CDs have connected or modified chemical groups, such as polymer chains and oxygen-based amino groups, on their surface [105,116]. Generally, the physicochemical properties, which include the structural, morphological, and optical properties, of most carbon-based nanoparticles (CNPs), including carbon dots (CDs), are typically ascertained using a wide variety of analytical techniques [18]. The Fourier transform infrared spectroscopy (FTIR) is a technique used to identify the surface functional groups that influence the solubility and environmental interactions of the nanoparticles. While X-ray photoelectron spectroscopy (XPS) investigates the chemical states and bonding environments of elements present on the surface, which is critical for assessing the extent of surface modification, X-ray diffraction (XRD) evaluates the crystallographic structure. These include microscopic techniques, such as high-resolution transmission electron microscopy (HR-TEM) and scanning electron microscopy (SEM), which offer a detailed examination of particle size and morphology, and in conjunction with elemental mapping, analyze surface texture and composition, as shown in Figure 8A. While X-ray photoelectron spectroscopy (XPS) investigates the chemical states and bonding environments of elements present on the surface [118], which is critical for assessing the extent of surface modification, as shown in Figure 8B. Fourier transform infrared spectroscopy (FTIR), which identifies the surface functional groups that influence the solubility and environmental interactions of the nanoparticles (Figure 8C). The thermal stability of CDs can be evaluated using thermogravimetric analysis (TGA) [119]. Atomic force microscopy (AFM) can be used to determine the roughness or thickness of CDs [119]. Additionally, Raman spectroscopy facilitates the differentiation between various carbon hybridizations, providing insights into structural defects and crystallinity (Figure 8E). In contrast, UV–Visible spectroscopy (UV-vis) serves to ascertain the optical absorbance properties, particularly in CDs (Figure 8D), which demonstrate significant absorption in the ultraviolet region [110]. The CD’s surface exhibits the n-π * transition at 310 and 355 nm, while the π to π * transition is specified by the light absorption peak at 230 nm [119]. X-ray diffraction (XRD) evaluates the crystallographic structure, revealing the degree of crystallinity in CNPs [34,36], as shown in Figure 8F. These techniques provide a comprehensive understanding of the behavior and potential applications of CDs in catalysis, drug delivery, sensors, and food packaging [18].

## 5. Recent Applications of Carbon Dots from Agricultural Extracts in Food Preservation

The application of CDs synthesized from agricultural extracts has garnered attention in recent years due to their eco-friendly nature, biocompatibility, and non-toxicity [122]. These carbon-based nanomaterials, derived from renewable and sustainable sources, have shown immense potential in various industries, including the food preservation industry, owing to specific biological properties [123]. Carbon dots from natural sources, such as agro-waste, exhibit excellent antioxidant and antimicrobial properties, making them effective in prolonging the shelf life of various food products [124,125]. For instance, CDs from lemons (*Citrus limon*) and onions (*Allium cepa*) demonstrate 90% radical scavenging activity at 100 μgmL^−1^ [126], while CDs from *Ananas comosus* had antimicrobial activity against common foodborne pathogens, such as *Bacillus cereus*, *Staphylococcus aureus*, and *Escherichia coli*, with a zone of inhibition of 28, 25 and 30 nm, respectively [127]. In addition, CDs integrated into cellulose nanofiber films extended the shelf life of tangerines and strawberries by more than 10 and 2 days, respectively [125]. Their small size, high surface area, and functionalization capabilities enable them to interact efficiently with food matrices, thereby maintaining food quality, reducing spoilage, and inhibiting microbial growth [123]. Recent advancements in this field demonstrate the feasibility of using CDs as additives in food packaging materials or coatings, offering a novel approach to sustainable food preservation methods. As a result, the integration of CDs from agricultural extracts in food packaging could significantly reduce post-harvest losses and promote environmentally friendly preservation strategies.

### 5.1. Carbon Dot Nanoparticles Applied as Antioxidant Agents

Antioxidants play a crucial role in neutralizing hazardous reactive free radicals that have the potential to cause oxidative stress and cellular damage [9]. Thus, the positive impacts in several fields, including food technology, medicine, and agriculture [128,129,130,131,132]. Certain minerals, vitamins, and enzymes are effective antioxidants that can minimize oxidative damage by neutralizing free radicals, also known as reactive oxygen species (ROS) [133]. The ROS are extremely reactive oxygen-containing molecules, such as singlet oxygen, alpha oxygen, superoxide, peroxides, and hydroxyl radicals, that are primarily produced from diatomic oxygen [134,135]. These oxygenated molecules can be toxic when present in excess in most biological systems, causing oxidative stress and harming cellular constituents, including DNA, proteins, and lipids [136]. However, at low levels, they can be helpful as defense agents against infections [134,135]. Studies of antioxidant properties are among the most researched biological activities of most manufactured chemicals and materials, including carbon dots, which have potential uses in agriculture and medicine [128,130,132].

Antioxidant agents are essential in food packaging to prevent oxidative degradation, which can cause rancidity, off-flavors, and the loss of nutritional value [137,138]. As a result, choosing the right antioxidant to include in the packing material is essential [123]. They help preserve fats, oils, and vitamins, such as A, C, and E, while maintaining the food’s natural color and reducing the formation of harmful compounds, such as peroxides [139]. By inhibiting lipid oxidation and protecting the food from spoilage, antioxidants extend shelf life and enhance safety [123]. Additionally, they safeguard active components in intelligent packaging systems, ensuring their effectiveness throughout the product’s life cycle [137].

**Figure 9:**
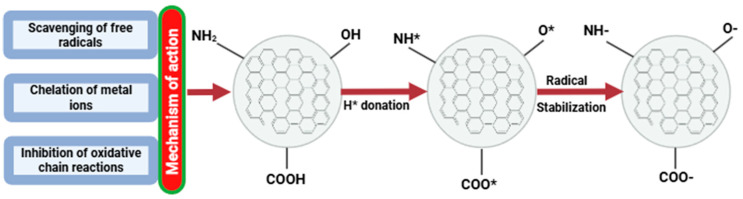
Mechanism of action of CDs through scavenging of free radicals, chelation of metal ions, and inhibition of oxidative chain reactions. The asterisks next to functional groups indicate unpaired electrons. Reproduced from [143].

Food oxidation and/or deterioration can be caused by photo-oxidation and photo-damage [140]. Therefore, several techniques have been adopted to guarantee food safety, including the development of suitable packaging technologies, including adequate antioxidant agents [25]. When CDs are infused into composite films, light-induced damage can be mitigated [122]. Several electron-donating groups, including hydroxyl and carboxyl groups, have been proposed as the cause of the strong antioxidant activity of CDs [141,142]. Recent studies have made the capacity of CDs to scavenge free radicals at the cellular level clear by using hypothesized mechanisms [95]. The main explanation for the antioxidant properties of CDs may be the metal ion chelation, inhibition of the oxidative chain reaction [143], production of ROS, and these highly reactive oxygenated free radicals can be suppressed [136], as shown in Figure 9. Through intracellular and extracellular interactions, these free radicals can be neutralized via hydrogen atom donation, electron transfer mechanisms, and the formation of reactive species from superoxide anion, nitric oxide, and hydroxyl from CDs [123]. Therefore, it is possible to argue that CDs have tremendous potential in various fields, particularly in food packaging, where oxidative stability is essential [123].

The most employed research methods to measure the antioxidant potential of CDs are 2,2′ -azino-bis (3-ethyl-benzothiazoline-6-sulfonic acid (ABTS+) and 2,2-diphenyl-1-picrylhydrazyl (DPPH) [144]. These useful techniques have been developed, and they have been demonstrated to be simple, rapid, and accurate in estimating antioxidant capacity [144]. The two primary techniques used to evaluate the total antioxidant capacity are generally acknowledged to be assays that employ the single electron transfer (SET) reaction, which is apparent by a color change when the amount of oxidant is reduced, and measurements utilizing the hydrogen atom transfer (HAT) approach [145]. Superoxide and hydroxyl radical scavenging rates are crucial markers of antioxidant activity [146]. As the concentration of Tea-CDs (TCDs) increased, so did their capacity to scavenge radicals, including superoxide and hydroxyl radicals. Tea-CDs demonstrated a notable ability to scavenge either hydroxyl or superoxide radicals, as seen by their respective IC50 values of 80 and 24.2 μg/mL [146].

The DPPH inhibition percentage of CDs was discovered to be dosage-dependent with Tea-based (TCDs), glutathione-based (GCDs), and grape (*Vitis vinifera*) pomace-based (P1CDs), demonstrating a percentage inhibition of 75%, 56%, and 46%, respectively, at a concentration of 375 µg·mL^−1^ [143]. On the other hand, the number of CDs required to reduce 50% of the initial DPPH was determined by calculating the average effective scavenger concentration (EC_50_). The EC_50_ values for TCDs, P2CDs, and GCDs were 50 μg·mL^−1^, 75 μg·mL^−1^, and 175 μg·mL^−1^, respectively, but P1CDs hardly achieved a 45% inhibition. These findings unequivocally demonstrate that TCDs have higher antioxidant capacity than either GCDs or CDs generated from pomace [143]. The decrease of DPPH• by the CDs may be attributed to the quenching of DPPH• by the transfer of hydrogen atoms from the carboxyl, hydroxyl, and/or amino groups. Resonance within the aromatic domains of CDs or the rearrangement of chemical bonds can delocalize the unpaired electrons from DPPH, being reduced to DPPH-H by absorbing an H, given by any of the surface groups [143]. In another study, carbon dots prepared from potato peels displayed significant antioxidant activity in ABTS+ and DPPH assays in a concentration-dependent manner [147]. The ABTS+ approach revealed a higher level of antioxidant activity than the DPPH method. This phenomenon can be attributed to the hydrophilicity properties of CDs, which have functional groups, such as hydroxyl groups, on their hydrophilic surfaces [147]. Other studies about the antioxidant capacity of carbon dots from plant extracts are summarized in Table 2.

### 5.2. Microbial Properties of Carbon Dot Nanoparticles

**Figure 10:**
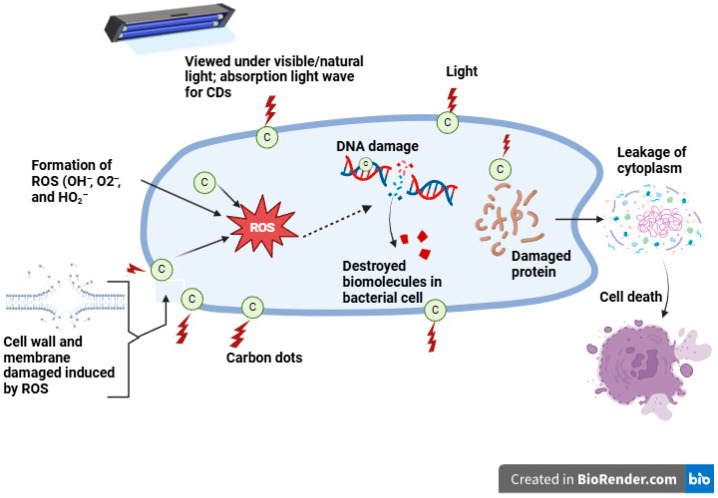
Antimicrobial activity of carbon dot nanoparticles on bacterial cell structure. Adopted and modified from [111].

Agriculture plays a significant role in countries around the globe [158]. However, significant losses in agricultural yield are caused by bacteria [159], nematodes [160], fungi [161], and other environmental microbes. Food pathogens produce a variety of diseases in commercially significant crops, which have a consequential impact on global trade [162,163]. Furthermore, these microorganisms can impact all stages of crop development, including sowing, production, and postharvest, and influence the quality and yield [158]. The lack of accurate, rapid pathogen detection techniques has financial implications that may result from product recalls and distribution delays brought on by tainted products entering the market [164]. Antimicrobial resistance (AMR) has increased significantly and grown progressively more hazardous in recent years due to the widespread and arbitrary application of antibacterial medications. Multidrug-resistant bacteria emerged because of an increase in AMR occurrences. Methicillin-resistant *Staphylococcus aureus* (MRSA) has become a prominent pathogen in nosocomial and community-associated illnesses because of the severe misuse of antibiotics [165]. Additionally, pathogens are accountable for the deterioration and degradation of various industrial products, foods, cosmetics, and medications, leading to large financial losses [166]. Hence, it is imperative to manage and limit their growth. Carbon dots have already gained popularity in the domains of biology and biotechnology because of their minuscule size, high surface charge, and configurable functional characteristics [167]. The primary applications of CDs in the field of food technology are the identification and detection of infections, antibiotics, pesticide residues, additives, and functional and nutritional elements [167]. When the CDs integrate with the target, effective ligands should be able to maintain the stability of the CDs’ chemical and optical characteristics while also conjugating with them securely [168]. Carbon dots have a unique fluorescence property, resulting in their application to detect harmful microorganisms that contaminate food. The on–off–on fluorescence detection approach makes high-sensitivity analysis and detection simple [169], to investigate the distinct advantages of CDs developed from papaya juice and use them as fluorescent probes for imaging *Bacillus subtilis* cells [170]. When activated at 488 (green) and 561 (red) nm, CD-labeled *Bacillus subtilis* cells produced a strong green and red fluorescence, demonstrating that the cells efficiently absorbed the manufactured CDs. Similarly, when excited at 488 and 561 nm, CD-labeled Aspergillus aculeatus produced green and red fluorescence images, indicating that the CDs were readily and successfully internalized by the bacterial cells [171]. This suggests that the CDs may be in the cytoplasm, particularly cocooned in the cell nucleus. As a result, the CDs made from pear (*Pyrus communis*) fruit have unique qualities, such as their microscopic size, multi-colored emission, and numerous organic groups on their surfaces, which make them potential probes for Al^3+^ ion detection and bacterial cell imaging [171]. In a study conducted by Mehta et al. CDs were synthesized through a hydrothermal process at 150 °C for 12 h using apple (*Malus* spp.) juice as a carbon source [172]. The resulting CDs were employed to detect the fungus *Magnaporthe oryzae* via endocytosis, as well as the bacteria *Pseudomonas aeruginosa* and *Mycobacterium tuberculosis*. These microorganisms were subjected to excitation at 405, 488, and 561 nm using 10 μg/mL CDs to obtain distinct fluorescence images. Kasibabu et al. involved the synthesis of carbon dots using green–yellow papaya (*Carica papaya* L.) juice via the hydrothermal method at 170 °C for 5 h [170]. Fluorescence imaging was performed to visualize *Bacillus subtilis* and *Aspergillus aculeatus*, which are responsible for secondary rot in various plants and food items, using 40 μg/mL CDs at 35 °C for 10 min with red (561 nm) and green (488 nm) excitation lasers in a confocal microscope. However, the synthesized carbon dots did not exhibit bacteriostatic properties against *Escherichia coli* at a concentration of 500 μg/mL [170]. These plant pathogens were detected using a 405 nm excitation laser in confocal fluorescence microscopy. Bukasov et al. synthesized carbon dots from palm (*Phoenix dactylifera* L.) fruit to investigate *Escherichia coli* via surface-enhanced fluorescence excited at 633 nm [173]. Carbon dots synthesized from *Ananas comosus* had antimicrobial activity screened against gram-negative bacteria *Pseudomonas aeruginosa*, *Bacillus cereus* (28 mm), *Staphylococcus aureus* (25 mm), *Escherichia coli* (30 mm), and *Vibrio cholerae* (14 mm) [127]. The precise mechanism by which CDs exert their antimicrobial effects is complex and remains inadequately understood [111]. However, it is believed that their antibacterial activity arises from several mechanisms, including the production of ROS, including O_2_^−^, OH^−^, and HO_2_^−^, the disruption of cell walls, the condensation of genomic DNA, and the release of cytoplasmic contents [174], as shown in Figure 10.

Carbon dots have surface functional groups, such as hydroxyl (-OH) and carboxyl (-COOH), and positive charges, which can affect antibacterial activity [175]. These groups could interact with the cell membranes of bacteria, resulting in structural damage and internal components leaking [175]. Furthermore, some CDs (such as those doped with nitrogen and phosphorus) have a positive charge that enables them to stick to the negatively charged bacterial cell walls, increasing their antibacterial potency [175]. According to Li et al., negatively charged CDs exhibit bacteriostatic properties, while neutral CDs exhibit minimal antibacterial effects [176]. Positively charged carbon dots generate greater quantities of ROS and function as more effective antibacterial agents. Nonetheless, ongoing investigations are aimed at elucidating the direct relationship between surface charge and ROS production [174]. Also, the surface charge of carbon dots plays a crucial role in their antibacterial efficacy [177]. Surface functionalization with amide and amine enhances their antimicrobial activity, damaging Gram-negative bacteria [178,179,180,181]. The CDs contribute to bacterial oxidative stress by generating hydroxyl radicals, further inhibiting microbial growth [182]. Moreover, CDs can generate ROS through light-dependent and independent reactions, imparting antimicrobial properties by inducing oxidative stress in bacteria and compromising their cell membranes via ROS [180,183]. Numerous studies have reported on the application of carbon dots (CDs) for food preservation [125,184]. Microbial contamination significantly threatens the long-term preservation of food items [167]. An additional advantage of employing CDs as food preservatives is their inherent non-toxicity [167]. Furthermore, CDs can be utilized in various capacities: (1) as components of metal oxide-based nanomaterials; (2) as heteroatom-doped carbon dots, typically sulfur- or nitrogen-doped; and (3) as functionalized carbon dots [95]. Other studies on the microbial application of carbon dots from plant extracts are summarized in Table 3.

### 5.3. Cytotoxicity of Carbon Dots

**Figure 11:**
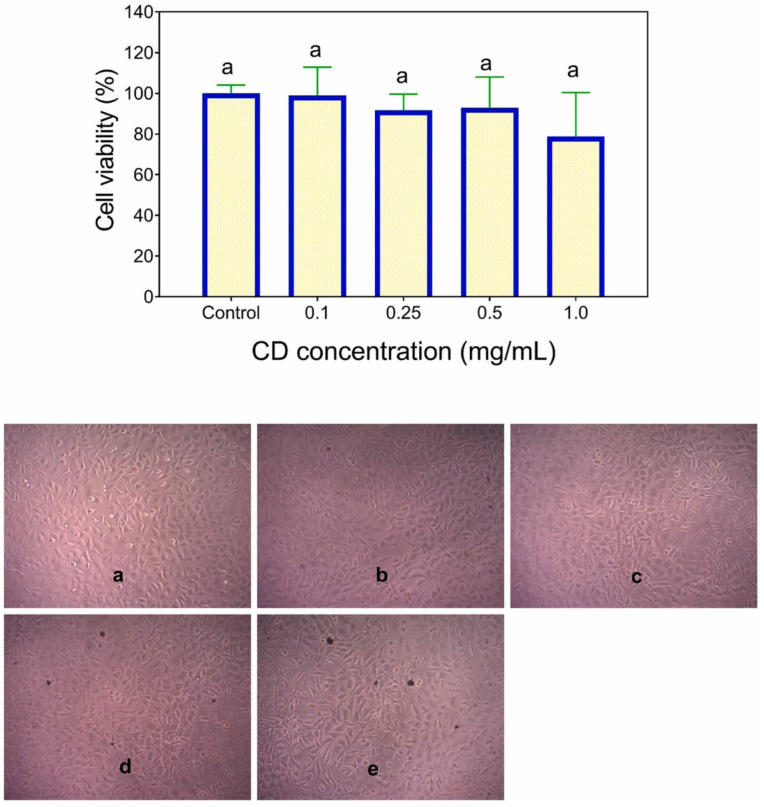
The photographs from optical microscopy and cell viability of mouse fibroblast L929 cells following a 72 h treatment with different concentrations of CDs from potato (*Solanum tuberosum*) peel. (**a**) Control, (**b**) 0.1, (**c**) 0.25, (**d**) 0.5, and (**e**) 1.0 mg/mL. The different letters indicate significant difference. Copied from [147], with permission from Elsevier.

Given their excellent stability, hydrophilicity, and biocompatibility, carbon dots exhibit lower cytotoxicity compared to metallic nanoparticles [189]. Comprehensive toxicological investigations, encompassing both in vitro and in vivo evaluations, are necessary to assess their possible long-term effects on human health and biocompatibility with other materials [190]. However, the cytotoxicity of CDs must be examined before they are applied in food packaging applications to ensure safety [119]. To date, there have been multiple publications documenting the cytotoxicity of CDs at the cellular level; however, the cell-damaging effect remains elusive [119]. Nevertheless, there is currently insufficient information on the toxicity evaluation of CDs [191], making it difficult to determine the exact nature, required dosage, and mechanism [192]. The problem with toxicity evaluations stems from material characterization, cell viability, drug release, and theoretical considerations [193]. As a result, quantification of CDs as food additives is crucial, since excess quantity can cause human health implications [2]. According to Liu et al., research on the cytotoxicity of CDs has shown that when irradiated with light, they can photodegrade and produce toxic compounds that could affect cancerous (HeLa and HepG2) and normal (HEK-293) human cells [194]. The nitrogen-doped CDs synthesized using kiwi (*Actinidia deliciosa*) fruit were used to evaluate whether N-CDs are biocompatible and cytotoxic to MCF-7 (Michigan Cancer Foundation-7) and L-929 (Lymphoblastoid-929) cells using the MTT (3-(4,5-dimethylthiazol-2-yl)-2,5-diphenyltetrazolium bromide) assay [63]. The N-CDs showed minimal anticancer capacity on MCF-7 human cells but not L-929 animal cells, with cell viability of less than 80% and 90%, respectively. The cytotoxicity of fruit-based CDs synthesized from kiwi, avocado, and pear was evaluated at 0.25–5.0 mg/mL on epithelial kidney cells (HK-2). The cells were cultured for 48 and 72 h, respectively. At concentrations greater than 1 mg/mL, cell viability slowly started to decline. Cell viability was generally higher for CDs synthesized from pears and lower for those from kiwis [195]. A toxicity assay on a live-cell line nematode model was used to evaluate the cytotoxicity of CDs produced from banana (*Musa* spp.) peel waste, which was assessed at concentrations ranging from 0 to 200 μg/mL, and the findings showed a decrease in viability of less than 5% [196]. Carbon dots synthesized from potato (*Solanum tuberosum*) peel using the hydrothermal method demonstrated low cytotoxicity when exposed to 0.1–1 mg/mL for 72 h, as >80% of mouse fibroblast L929 cells survived, as shown in Figure 11 [147]. The interaction of CDs with biological systems varies depending on the cell system under investigation and the cytotoxicity assay approach. These interactions depend on variables such as surface charge, surface coating, size, etc [119]. To guarantee the safe application of CDs into food applications, various methods have been developed or proposed an array of mitigating techniques, such as functionalizing and modifying the surface, employing natural precursors, and regulating the size of CDs using various synthesis and purification techniques [197]. Applying an appropriate precursor and reaction conditions is an effective approach to mitigate potential risks associated with CDs tailored for specific applications in the food industry [197].

## 6. Application of Carbon Dots in Food Packaging

The World Health Organization (WHO) has released alarming figures that underscore the urgent need for improved food packaging systems to combat foodborne illnesses and spoilage. In 2015, nearly 600 million people were affected by food contamination, resulting in 420,000 deaths [198,199]. Diarrheal diseases, primarily caused by consuming contaminated food, accounted for 550 million illnesses [200]. These findings highlight the urgency for new packaging solutions and the vulnerability of global food safety. In addition to preserving food quality, effective food packaging is essential for minimizing contamination throughout distribution, storage, and transportation, reducing the risk of foodborne infections, and helping alleviate food shortages [201,202].

Food product shelf life can be increased, and spoilage and waste can be greatly decreased by integrating antimicrobial and antioxidant agents into packaging materials. Intelligent and active packaging are examples of advanced packaging technologies that offer real-time food quality monitoring and preservation, ensuring safer consumption and minimal environmental impact [203]. Adopting innovative packaging solutions will be essential in preventing foodborne diseases, reducing food spoilage, and tackling the issue of global food security as the global population continues to expand and food demand increases. Recent developments in nanoscience have made nanotechnology a promising approach to food safety sensing, with the ability to address numerous issues regarding food safety [203].

Traditional synthetic plastics, such as polypropylene (PP), polyethylene terephthalate (PET), high-density polyethylene (HDPE), low-density polyethylene (LDPE), polyvinyl chloride (PVC), polystyrene (PS), and thylene vinyl alcohol copolymer (EVOH), have played a significant role in the packaging sector due to their low cost, excellent water and light barrier properties, and durability [140]. However, these materials are non-biodegradable since they are made from nonrenewable petroleum resources. Moreover, the packaging industry has been reported to generate 6.3 billion metric tons of plastic waste annually, from products including trash bags, shopping bags, food packaging, and electronic packaging, with only 9% being recycled, and 79% of it accumulating in soil, while the rest are found in the sea. By 2050, 12 billion metric tons of plastic will be discarded into landfills if current trends continue [204]. Furthermore, heating polymers, such as PVC, can release toxic chemicals, such as dioxins and furans, which contribute significantly to environmental pollution [205].

To address these impediments, bio-based and biodegradable polymers derived from starch, polycaprolactone (PCL), polybutylene succinate (PBS), poly lactic acid (PLA), polyhydroxyalkanoate (PHA), and cellulose-based polymers have gained attention as sustainable alternatives [206]. The application of naturally biodegradable materials in active packaging applications is growing due to their environmental benefits, alongside their mechanical and processing properties [140]. The market for biodegradable plastics is projected to expand at a 15.1% compound annual growth rate (CAGR) from 3.02 billion USD in 2018 to 6.12 billion USD in 2023 [140], demonstrating how the packaging industry is shifting toward more sustainable solutions. As a result, carbon dots have emerged as promising nanomaterials for food packaging. These nanomaterials provide several benefits, including minimal toxicity, excellent compatibility with biopolymers, and strong antioxidant and antimicrobial properties [124,207]. The incorporation of CDs into biodegradable polymers can enhance the mechanical, water barrier, and UV barrier properties of the packaging materials [122,125]. Due to these functional improvements, CDs provide a practical solution for addressing food contamination and extending postharvest shelf life [124], while also advancing the development of eco-friendly packaging materials.

### 6.1. UV Barrier Properties of CDs on Polymers

Food packaging films serve multiple crucial purposes, including transparency and UV protection [208]. Transparent packaging facilitates the consumer’s evaluation of the visual quality of the products. Furthermore, transparent packaging film can transmit light, making food quality susceptible to photodegradation. Thus, photochemical reactions caused by certain light wavelengths are prevented, thereby extending the shelf life of the product [119]. In recent years, nanomaterials such as CDs have been incorporated into packing materials to scatter and absorb light. Carbon dots can be integrated into food packaging materials to protect food from harmful UV radiation due to their high scattering and adsorption qualities, which permit them to immediately convert UV photons into heat [209].

Packaging materials enriched with CDs can effectively block short-wavelength UV radiation while permitting the passage of long-wavelength visible light and significantly enhancing the UV-barrier properties of the packaging film without compromising its transparency [119]. For instance, Sul et al. evaluated the optical properties of chitosan-gelatin composite films by analyzing the light transmittance at wavelength 660 nm (T_660_) to evaluate transparency and wavelength 280 nm (T_280_) to assess UV-barrier effectiveness, to shed light on the plastic film’s potential applications as a protective packaging material [208]. The incorporation of CDs (7.5%) into the chitosan + gelatin film reduced T_280_ (to 0.07%), indicating that adding CDs provides complete UV protection at the smallest loss of transparency [208]. Similarly, Khoshkalampor et al. examined the transparency of gelatin-based films and demonstrated that adding CDs to films resulted in a significant reduction in their transparency [210]. However, films with higher CDs concentration demonstrated a significant decrease in UV-A and UV-B transmission rates when compared to control films, such as gelatin, PG/gelatin, and A3 [210]. Chitosan nanocomposite hydrogel films enriched with CDs were found to have better UV-visible blocking. Transmittance for CH-CD4 was up to 20% lower than that of CH hydrogel film in the 300–600 nm wavelength range [24]. The incorporation of CDs into film matrices significantly improved their UV barrier capabilities, which are crucial for food packaging applications to preserve food quality. Moreover, CD-incorporated composite films improve UV protection and barrier qualities in addition to imparting antioxidant and antimicrobial properties due to the strong interaction that forms between CDs and the polymer matrix through a hydrophilic bond [147,211].

### 6.2. The Effect of CDs on the Mechanical Properties of Nanocomposite Films

Food products must be packaged in films that possess sufficient mechanical durability and structural integrity for effective protection and preservation of quality by preventing breakage or leakage encountered during the value supply chain [208]. Consequently, biopolymers have poor mechanical properties, which can hinder their commercial application and often require nanofillers to overcome this impediment and retain their structural integrity during storage and transportation [210]. Evaluating key parameters, such as thickness, tensile strength (TS), and elongation at break (EB), is crucial to gain insight into their flexibility, strength, and resistance to mechanical pressure of the food packaging materials [203]. For instance, incorporation of CDs into cellulose nanofiber-based polymer matrix influenced the thickness of the film in a concentration-dependent manner, rising from 0.0225 mm to 0.0254 mm, caused by the increase in solid content [203]. The interaction between the polymer matrix and nanofiller has a significant impact on the TS of food packaging materials, as high compatibility facilitates the uniform dispersion of nanofillers [119]. Smaller CDs (<10 nm) disperse easily in the polymer matrix nanocomposites, preventing clumping or agglomeration, resulting in uniform biopolymers [212,213]. In contrast, larger CDs tend to aggregate more readily and form clusters that are difficult to disperse in the polymer matrix, affecting the mechanical properties of polymer nanocomposites [213,214]. Therefore, maintaining the structural integrity of the polymer matrix is fundamental to ensure there is strong dispersion and hydrogen bonding between the different constituents [210]. This was observed in films formulated using Persian gum (PG), gelatin with carboxylic and amino groups, and hydroxyl groups of CDs. The addition of CDs to films at a 5% dry weight enhanced their TS when compared to the PG: gelatin film [210]. However, when more than 5% of CDs were added to the films, the tensile strength significantly decreased (*p* < 0.05), presumably because of the aggregation caused by CDs [210]. Higher concentrations of hydrophobic nanomaterials may cause aggregates to form in the polymer matrix, disrupt the structured molecular arrangement of the polymer matrix, and, given that the nanofillers are not dispersed uniformly throughout the polymer matrix, the film strength is reduced [215]. This phenomenon was observed in the study carried out by Bao et al. in which the addition of CDs to gelatin film with 10 % CDs (G/10CD) reduced the tensile strength by 3 MPa compared with gelatin (G) film, coupled with an increase in elongation at break (170%) [122]. Therefore, it is necessary to apply nanofillers with multifunctional properties, such as surface hydrophobicity [208], antibacterial activity, and biocompatibility [119].

### 6.3. The Effect of CDs on Water Vapor Permeability of Nanocomposite Films

Food quality deteriorates during storage and transportation due to moisture movement between the food product and the external environment [208]. Food items with high moisture content need to be stored in a dry environment to prevent water inside the food from evaporating; this necessitates a certain level of water barrier packaging materials [216]. The capacity of the food packaging material to permit water diffusion between food and the external environment can be measured by water vapor permeability (WVP) [210]. As a result, low WVP packaging films are recommended to maintain food quality and increase shelf life [208]. Water vapor transmission through a film involves a series of processes, namely (1) water vapor adsorption on the surface of the film, (2) water vapor dispersion across the film, and (3) evaporation on the opposite side of the film [119]. The water vapor diffusion route length is determined by the structural arrangement of the polymer, which also influences the water vapor migrating through the film [119]. When CDs (3 wt% based on polymer) synthesized from banana peels were incorporated into the neat chitosan + gelatin film, there was no significant change in the WVP, which was 1.24 × 10 Pa s [208]. Similarly, the addition of CDs to the Persian gum (PG) + gelatin films displayed no significant change to the WVP [122]. However, when 2.5 and 5% of CDs were added, WVP values decreased insignificantly (*p* > 0.05), but when 15% of CDs were added, WVP values increased in contrast to Persian gum + gelatin films (*p* > 0.05). The initial decrease in WVP and water vapor diffusion could be explained by a tortuous pathway created by cross-linking between Persian gum, gelatin, and CDs; the subsequent increase may be caused by the abundance of hydroxyl groups in CDs, which give them their hydrophilic properties [210]. Optimization is necessary since adding CDs to packaging film has the limitation of reducing the water vapor barrier properties.

### 6.4. The Effect of CDs on the Antioxidant Properties of Nanocomposite Films

Food deterioration is primarily caused by oxidation. Although extensive research has been conducted to reduce oxidation, the most prevalent approach is still adding antioxidants directly to food [140]. Consequently, there are two limitations to this approach: when antioxidants interact with food components during processing, their activity may be reduced or impeded, and the quality of the food deteriorates rapidly upon consumption of nutrients contained in the food item [217]. To address these limitations, researchers conducted research into active food packaging approaches. Numerous investigations have been conducted on food packaging materials with antioxidant agents integrated into the polymer matrix or on their surface. The three primary migratory actions of antioxidant agents are distribution between interfaces, transferring from packaging materials to the food surface, and diffusion inside the polymer structure [218]. Because they can reduce oxygen in food systems, antioxidants can effectively delay the oxidation of lipids and the denaturation of proteins. They inhibit the oxidation process and prevent it from proceeding [218]. Moreover, they can render the enzyme ineffective in catalyzing the oxidation reaction by suppressing or deactivating its capacity to function [218]. When added to active packaging, antioxidants should be highly active at minimal amounts, stable, nontoxic, and highly permeable, and they should not compromise the quality of the food [218].

There have been attempts to incorporate polyphenolic chemicals, plant extracts, or essential oils as antioxidant agents into packaging materials [219]. Since CDs have exceptional antioxidant activity, good antibacterial qualities, low toxicity, and high compatibility with most biopolymers, they are attractive candidates for use as nanofillers in active food packaging films [125,184]. When 5% and 10% CDs were added to cellulose nanofiber composite film, the DPPH radical scavenging activity was 60% and 80%, respectively. After adding 10% CDs, the DPPH radical scavenging activity did not rise further. The composite film with cellulose nanofibers demonstrated an increase in ABTS radical scavenging activity as the concentration of CDs increased. As a result, 10% CDs were the optimum concentration for additional research on the cellulose nanofiber composite film [122]. At 734 nm and 517 nm, the distinctive ABTS and DPPH absorbance bands progressively disappeared as the concentration of CDs increased [157]. Likewise, Sul et al. investigated the antioxidant activity of the chitosan + gelatin composite films using the DPPH and ABTS methods [208]. Tammina et al. used carboxymethyl cellulose + agar-based films enriched with CDs doped with nitrogen, which demonstrated strong antibacterial activity and high antioxidant levels for DPPH (12.7%) and ABTS (67%) [205]. The chitosan + gelatin film demonstrated slight radical scavenging activity of 10% and 20% for DPPH and ABTS, respectively. However, chitosan + gelatin film enriched with CDs had strong antioxidant activity, exhibiting 74.5% DPPH and 100% ABTS radical scavenging activity [208]. Due to the OH groups on their surface, the CDs are more widely distributed in the ABTS aqueous solution, increasing the ABTS method’s activity. Through the reduction capability of active antioxidants, they can effectively delay the oxidation of lipids and denaturation of proteins by reducing the amount of oxygen present in food systems [140]. Additionally, they interrupt the oxidation chain reaction, prevent the oxidation reaction from proceeding, and inhibit or deactivate the enzyme’s activity so that it cannot catalyze the oxidation reaction [140]. Films enriched with CDs that demonstrate excellent antioxidant activity are highly recommended for application as active packaging films to prevent food from oxidatively degrading during storage and transportation [184].

### 6.5. Antimicrobial Properties of CDs on Polymers

In active food packaging applications, antimicrobial activity is the most used functional feature to ensure food safety and prolong shelf life by preventing the proliferation of pathogenic microorganisms [119]. Recent research has shown that CDs exhibit strong antibacterial activity against bacteria and fungi due to their surface charge, size, and morphology. The antimicrobial activity of films is assessed using techniques such as viable colony count, well diffusion, disk diffusion, and optical density. Synergistic effects with CDs are frequently added to biopolymer matrices, such as chitosan, carrageenan, and starch, to enhance their antibacterial properties [29]. Sul et al. formulated composite films using chitosan and gelatin, which displayed minimal antimicrobial activity screened against *Escherichia* coli and *Listeria monocytogenes*, due to the antimicrobial activity from chitosan [208]. In contrast, composite films enriched with CDs inhibited the microbial growth of *Listeria monocytogenes* and *Escherichia coli*, in addition to chitosan + gelatin films. The increase in antimicrobial activity of the chitosan + gelatin + CDs composite film was due to the antimicrobial activity of CDs, which is related to the slow release of CDs from the composite film [208]. Also, carboxymethyl cellulose + agar films with 8% CDs doped with nitrogen prohibited the proliferation of *Escherichia coli* and *Listeria monocytogenes* [205]. To ascertain how well CD-containing polymer matrices function in actual food, more research is necessary [141]. The CDs demonstrated superior efficacy as a multipurpose nanomaterial with durable antioxidant and antibacterial properties. Due to their exceptional functional qualities, high biocompatibility, and minimal toxicity, CDs can be employed as multifunctional nanofillers in food packaging applications. Thus, many studies have been carried out in which CDs were used as an additive for active packaging purposes. Nonetheless, more examples of the biological studies of CDs for food packaging materials, the various preparatory approaches, and conditions alongside key findings are summarized in Table 4.

## 7. Application of Edible Coatings Enriched with CDs for Food Preservation: Enhancing Shelf Life and Food Safety

Foodborne infections can lead to significant loss of fruits and vegetables at any stage during the post-harvest handling process. In addition to the financial implications, consuming contaminated produce poses severe health risks to consumers [3]. Although chemical treatment with synthetic fungicides remains a common method for managing post-harvest diseases, it has several limitations, such as environmental pollution, side effects on non-target species, and the emergence of fungicide-resistant pathogens [234]. Furthermore, several fungicides have been prohibited by EU regulations due to these concerns, highlighting the need for alternate disease prevention strategies that may mitigate disease incidence while also reducing risks associated with the environment [234].

Fruits and vegetables are particularly vulnerable to microbial spoilage due to their high moisture content and ongoing biological activities after harvest, rendering them susceptible to microbial contamination and rapid degradation [235]. Ensuring quality preservation during transportation and storage is crucial from a technological and economic perspective [184]. Recent advancements in biopolymer-based active packaging materials, such as biopolymer-based active thin films and edible coatings, have shown great promise in mitigating microbial growth and preserving the quality of perishable produce. These biopolymer-based active thin films and coatings protect from physical and chemical modifications and act as UV barriers, reducing food oxidation caused by UV light exposure [236].

In this context, nanomaterials such as CDs offer an innovative solution for food packaging materials owing to their biological properties mentioned in the previous section [184]. Therefore, incorporating CDs into food packaging materials can help reduce spoilage, prevent microbial contamination, and extend the shelf life of perishable foods [126]. This integration further endorses the shift toward eco-friendly, sustainable packaging solutions, providing a viable alternative to conventional chemical preservatives and synthetic fungicides.

Edible coatings can increase shelf life by regulating the gas exchange (such as CO_2_ or O_2_) between the fresh food and its environment, which delays ripening and spoilage and regulates respiration [125]. Numerous techniques for applying these materials to food items have been reported in the literature, and these include spraying, panning, dripping, dipping, and fluidized bed coating [204]. Fresh produce can be submerged or sprayed in the film-forming solution, thereby creating a thin protective active film layer around the food surface [219].

CDs have strong antibacterial, antifungal, and antioxidant activity, and display excellent compatibility with a wide variety of biopolymers, making them a viable option for creating food packaging films as functional nanofillers [184]. Information pertaining to the food product is crucial for optimizing the different coating formulations, knowledge of targeted pathogens, and selecting a suitable preservation method. A statistical technique known as response surface methodology is implemented to optimize formulations by determining the most optimal combination of ingredients for films and coatings to reduce experimental runs [237]. However, stand-alone films have primarily been used in vitro to assess the optimization of coating formulations incorporating food additives with antibacterial activity [238]. Many reports have been made on the use of packaging materials with embedded CDs on various food items.

### 7.1. UV Barrier Capabilities of CDs Enhance the Shelf Life

**Figure 12:**
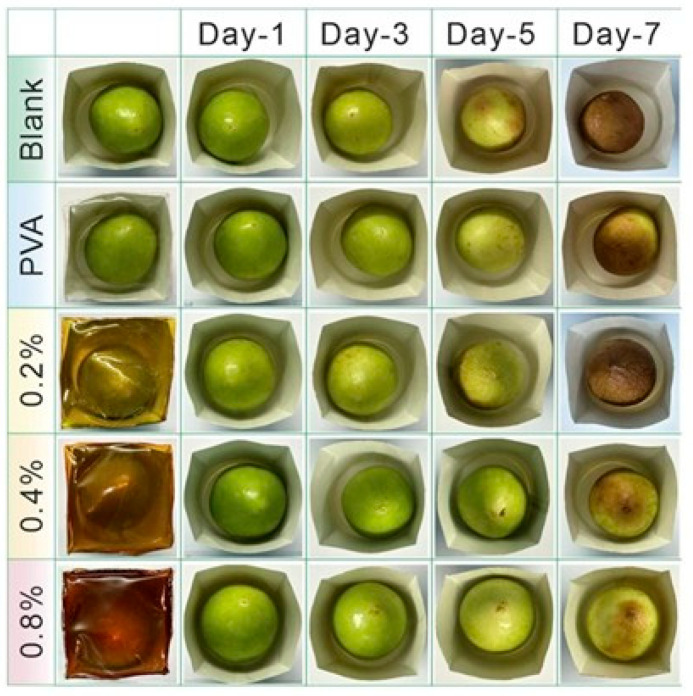
Photographic images of jujubes during 7-day storage: Blank, PVA, DEF-CDs/PVA- 0.2 %, DEF-CDs/PVA-0.4 % and DEF-CDs/PVA-0.8%. Reproduced from [240] with permission from Elsevier.

Microorganisms, light, and oxidation processes are typically the primary causes of food deterioration since UV radiation often accelerates the degradation process by reducing nutrients, stimulating microbial growth, and altering the color or texture of food [239,240]. Fruits and vegetables need to be protected from ultraviolet (UV) radiation since many essential nutrients are lost when exposed to UV radiation [228]. UV degradation can be significantly reduced by incorporating UV-blocking agents, such as CDs, into the composite films. The biocompatibility, fluorescence, and UV-barrier properties of CDs (<10 nm) make them desirable nanofillers that are ideal for food preservation [241]. For instance, Patil et al. prepared polyvinyl alcohol/waste tea residue CDs (PVA@WTR-CDs) composite film and used grapes as a model fruit [228]. The experiment was conducted under a UV lamp for 30 hr in two sets. The grapes were arranged in paper teacups A, B, and C. Cup A was unwrapped and served as a control. However, cups B and C were wrapped in pristine PVA film and PVA@WTR-CDs composite thin film and monitored in a UV chamber at different intervals up to 30 hr. After 15 hrs of exposure to UV light, there were significant changes in color, size, and shape of the grapes in cups A and B as they appeared to be more brownish and shriveled compared to those in cup C. The results indicate that PVA@WTR-CDs composite films have better UV blocking properties and are practically applicable to food packaging. Duan et al. developed an active food packaging film using polyvinyl alcohol doped with CDs from diethyl ferulate (DEF-CDs/PVA) and investigated the film’s protective effect on jujubes, which are susceptible to deterioration and browning caused by photooxidative damage [240]. The fruits were wrapped in the films and subjected to UV radiation (at 18 W) for 7 days, and visual appearances were captured using a digital camera, as shown in Figure 12. The findings demonstrate that after jujubes were wrapped with DEF-CDs/PVA films, the brown stain was significantly reduced. On the 7th day, samples treated with 0.8% DEF-CDs/PVA film maintained their yellow–green color, whereas the jujubes in the blank control and PVA film groups went entirely brownish black. The color change is caused by the reactive oxygen species (ROS) produced by jujubes under ultraviolet radiation, causing chlorophyll degradation [240]. The process of chlorophyll degradation was significantly reduced due to DEF-CDs/PVA UV barrier property [240].

### 7.2. The Effects of CDs on Microbial Count

The most significant factor contributing to food spoilage during handling and storage is the increase in the microbial load [242]. Chitosan coating solutions with CDs improved the antimicrobial activity by forming a protective layer against mold, yeast, and the total number of colonies [243]. For instance, carbon dots were integrated into kelp + chitosan edible coating solution and applied to fresh-cut cucumbers at concentrations of 0%, 1.5%, 3%, and 4.5% [243]. The cucumbers were subsequently packaged and kept at 4 °C for 15 days. The investigation focused on the impact of CDs + chitosan coating on foodborne pathogens and the fresh-cut cucumber quality when stored in a changed atmosphere. Strong hydrogen bonds were created by the interaction of CDs with chitosan. The increase in CDs concentrations was associated with an increased inhibition zone of CDs + chitosan coating against *Escherichia coli* and *Staphylococcus aureus* [243]. Additionally, when fresh-cut cucumbers were stored in a modified-atmosphere package, the CDs + chitosan coating prevented the growth of all colonies, mold, and yeast. Strawberries (*Fragaria ananassa Linnaeus*) were treated with LCDs (lemon carbon dots) and OCD (onion carbon dots) to reduce microbial proliferation and extend shelf life, as shown in Figure 8. The rate of decay for OCDs was 11%, LCDs was 40%, and the control was 96.65% on the same day [126]. Similarly, strawberries coated with Gelatin + 1.5% R-CDs had a decay rate of roughly 4.17%, whereas uncoated strawberries had a decay rate of 75% on day 8 of the storage period [235]. The antimicrobial activity of CDs can prevent microbial spoilage, maintain texture, and prolong the shelf life of fresh produce. However, to completely comprehend the long-term implications and the optimal concentration of carbon dots in food preservation.

### 7.3. The Effects of CDs on the Physiological Properties of Food Products

**Figure 13:**
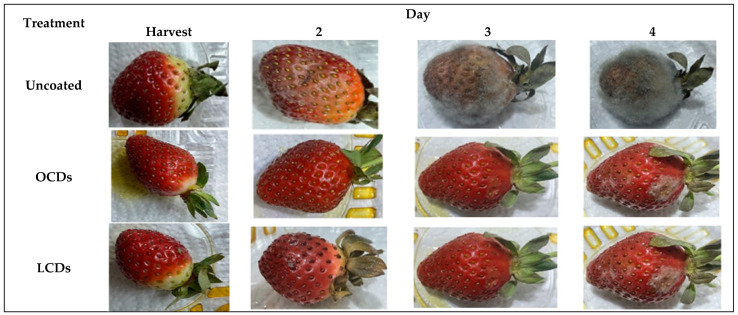
Visual image of strawberries packaged with LCDs and OCDs during the 4-day storage period. Adapted and modified from [126] with permission from Elsevier.

The respiration rate is a measure of metabolic activity change, which may indicate the decline in fruit quality [244]. Several variables affect respiration rates, such as temperature, storage conditions, type of fruit, cutting process, and maturation stage [245]. Fresh-cut fruits that have been cut have damaged tissues. As a result, metabolic activity accelerates as the cells seek to regenerate. This frequently leads to a higher respiration rate and a rapid deterioration rate than when the fruit is whole [245]. Carbon dots can help preserve cellular activity for an extended period by improving membrane stability and reducing the respiration rate of the fruit. For instance, in carboxymethyl chitosan coating formulation with CDs applied on fresh-cut pears (*Pyrus communis*), the authors demonstrated that chitosan and *Pleurotus eryngii* carbon dots (CS/2%PER-CDs) coating solution effectively reduced the respiration rate (51.67 mg CO_2_/kg⋅h) and ethylene production rate (0.75 μg/kg⋅h), thereby maintaining the quality of fresh-cut pears (*Pyrus communis*) [245]. Another study reported that cucumbers treated with ultrasonic (US), chitosan (CH), carbon dots (CDs) coating, and US + CDs significantly (*p* < 0.05) reduced respiration rates compared to the untreated group, as shown by the decrease in respiration rate (4.67 mg kg^−1^ h^−1^ CO_2_) of samples treated with US+CDs. The high biocompatibility of chitosan and CDs containing abundant functional groups might be the contributing factor, along with the increasing surface crystallization degree and roughness of the films [246]. The rate of respiration was decreased by altering the endogenous gas exchange and creating a microenvironment on the sample surfaces [244]. These findings demonstrated how a combined application of US and CDs coatings reduced the respiration rate of cucumber [243]. The primary factor affecting the storage life and quality of the fruit appears to be weight loss, which is related to the production of carbon dioxide and water loss during the respiratory process of fruits, accelerating spoilage and decreasing their marketability [244]. The weight loss of cucumbers (*Cucumis sativus*) treated with CDs/CH coating displayed the lowest weight loss at 7.82% at the end of storage trials compared to control samples [243]. When CDs were incorporated into a chitosan coating solution, the degree of surface crystallization increased, creating a good barrier that reduced water vapor permeability [247]. Also, CDs (4.5%) + chitosan coating significantly inhibited peroxidases activity, decreased water mobility, reduced weight loss, firmness, and soluble solids losses, as well as ascorbic acid content and flavor deterioration [243]. Similarly, Riah et al. formulated carboxymethyl cellulose with CDs 3.0 wt% and applied the coating solution on lemons (*Citrus limon*) [23]. The treatment with CDs retained their original flavor and color, and the surface of the lemons displayed no signs of mold growth. Carbon dots help prolong the shelf life of fruits by minimizing the transpiration rate, reducing weight loss, enhancing protective layers, improving cellular integrity, and producing antioxidant and antimicrobial effects. Nevertheless, further research is required to optimize their application in food preservation and ensure long-term efficacy and safety. Various applications of carbon dot-based packaging material on different food products, alongside their respective findings and impacts, are summarized in Table 5 and Figure 13.

## 8. Challenges and Future Directions

Numerous in vitro and in vivo research investigations monitoring potential health effects from exposure to nanomaterials in agri-food-related products are available in the literature [250]. The safe manufacturing and management of nanomaterials for use in the food sector is a major concern for governments across the globe [251]. Important measures to be considered while evaluating the potential risks and safety regulations of nanotechnology employed in food products are legislation, recommendations, and guidelines issued by legal authorities [252]. The primary authorization bodies in Europe and the US that oversee regulations and guidelines pertaining to nanomaterials in food are the European Commission (EC), European Food Safety Agency (EFSA), Food and Drug Administration (FDA), Environmental Protection Agency (EPA), International Standard Organization (ISO), Organization for Economic Cooperation and Development (OECD), World Health Organization (WHO), and Scientific Committees and Agencies [250]. Conventional regulatory protocols specify that nanoparticles can be identified by their size, stability, and chemical composition, and surface properties are crucial in determining potential interactions and persistence of the materials in the body, hence providing information for risk assessment [250].

Even though CDs derived from fruit extract are usually thought to be less toxic than those made from synthetic precursors, comprehensive toxicity and biocompatibility investigations are required, particularly for applications in biomedicine, such as drug delivery, food packaging materials, bioimaging, and biosensing [253]. Moreover, CDs have great potential in food preservation due to their antibacterial and antioxidant qualities [141], photoluminescent [254], and real-time detection [255], even though they still lack sufficient multiplex sensing capabilities. Advances in sensor technology and microfluidics may result in more effective food safety management systems due to current investigations into CDs as food preservatives and their broader application in the food sector [256]. Surface functionalization is one of the most important factors influencing the biological uses of nanomaterials since it may identify intracellular trafficking, cytotoxicity, and cellular uptake pathways [257]. However, more research is required to comprehend the mechanism of action of CDs in food items, particularly the interaction with the food matrix and prolonged exposure at low concentrations to develop strategies for regulating their release [257,258]. Animal models exhibit a significantly shorter lifetime compared to humans in chronic long-term exposure scenarios, which is one of the restrictions of nanoparticle risk assessment [259]. Research on long-term low-dose nanoparticle exposure could offer significant new insights into the long-term adverse effects of nanoparticles, especially with reference to standardized toxicity testing procedures and dosage evaluations [259].

The process of synthesizing CDs with uniform size distribution can be challenging. Because plant extracts naturally differ in content, synthesizing CDs from them may result in variations in size distribution, morphology, QY, fluorescence, and surface chemistry [89,111], which could hinder their biological applications, especially in vivo. The influence of reaction conditions, such as the nature and concentration of precursors, temperature, time, and pH, on the performance of CDs should therefore be systematically investigated to understand the mechanism of CDs formation [111,260]. Another challenge in using nanostructures on a commercial scale is their low production rate [261]. The lack of a systematic and scalable synthesis technique for producing high-quality CDs with desirable physicochemical properties has been addressed to increase the rate of production to make them economical [262]. These would include machine learning-driven methods to screen CD precursors and predict their physicochemical properties [263]. Researchers can rapidly identify the most efficient and effective method to produce CDs courtesy of machine learning (ML) algorithms, which have become an effective tool for synthesis optimization [263]. For instance, Dager A et al. used various ML models to describe the synthesis of the photoluminescence mechanism in the CDs [264]. These models included multivariate curve resolution (MCR-ALS), auto relevance determination (ARD), non-negative matrix factorization (NMF), soft orthogonal constraint (SO), and principal component analysis (PCA). Senanayake et al. used an artificial neural network (ANN) to synthesize CDs that have specific emission properties and evaluate how the synthesis variables affect CDs’ properties [265]. While CDs synthesized from renewable resources are promising, their complex synthesis procedures, expensive equipment, low production yields, and extreme conditions (strong antioxidants, corrosive agents, strong acids, such as H_2_SO_4_, HNO_3,_ etc.) may hinder their industrial application [266]. Combining CDs with smart biopolymers that respond to environmental stimuli (such as pH, temperature, and gas presence) may result in intelligent packaging that provides real-time freshness indicators by changing color or fluorescence in reaction to food degradation [267]. Innovative methods, such as electrospinning and layer-by-layer for manufacturing, enable the accurate dispersion of CDs in biopolymer fibers or multilayered films, which results in localized sensing zones inside the package, improved mechanical performance, and adjustable release behaviours [268]. Therefore, more research is required to develop an efficient strategy to optimize large-scale, eco-friendly production [89,266]. Despite all those obstacles, carbon dots have unexplored, immense potential in the food industry.

## 9. Conclusions

This literature review has demonstrated that carbon dots made from agricultural waste are not only a viable and affordable alternative but also possess significant functional characteristics, such as photoluminescence, biocompatibility with polymers, strong antibacterial activity, improved barrier properties of food packaging films, and extended shelf life of horticultural crops. Evaluating carbon dots produced from agricultural waste extraction offers a viable and sustainable approach to current problems with food safety and shelf-life extension. Nanotechnology and food science benefit from this multidisciplinary approach, fostering innovation and novel discoveries. Reducing food waste by extending food goods’ shelf life and preventing spoilage can save manufacturers and consumers money. Furthermore, the possibility of combining these carbon dots with other preservation techniques provides new opportunities for more effective and thorough food safety policies. Nonetheless, importantly, the review identifies critical gaps in toxicity evaluation and the lack of data on nanoparticle migration in food contact scenarios. These must be addressed for regulatory approval and commercial adoption. Furthermore, to enable broader implementation of this technology, several limitations still need to be overcome, such as scalability and safety regulatory approval. Thus, new studies should concentrate on optimizing synthesis techniques, maintaining uniformity in quality and safety, and investigating the entire range of applications in the food sector. This area holds great potential for improving food preservation technology, guaranteeing food security, and fostering sustainability with continued research and development.

## Figures and Tables

**Table 1:** The carbon source from agricultural extracts, production techniques, quantum yield, and photoluminescence of synthesized carbon dots.

Carbon Source	Type of Carbon-Based NPs	Production Techniques	Size	Key Findings QY (%)	Reference
Banana (*Musa* spp.) peels	CDs	Microwave treatment 500 W for 20 min	2.2 nm	NR	[56]
Banana (*Musa acuminata*.) Juice	CDs	150 °C for 4 h.	3 nm	8.95%.	[57]
Blueberry (*Vaccinium* spp.)	CDs	Liquid nitrogen-assisted, for 30 min	NR	NR	[58]
Carrot (*Daucus carota*)	CDs	Hydrothermal carbonization for 5 h at 180 °C	2–7 nm	11.5%.	[59]
Cashew Nut Skin (*Anacardium occidentale*) Waste	F-CNDs	Hydrothermal, at 200 °C for 24 h.	2.5 nm	15.5%	[49]
Dragon (*Hylocereus undatus*) fruits	N-CDs	Hydrothermal carbonization, 180 °C for 12 h	2.5 nm	NR	[60]
Dwarf banana (*Musa* spp.) peel	N-CDs	200 °C for 24 h.	4.0 nm	23%.	[53]
Grapefruit (Citrus × paradisi) peel	CDs	Hydrothermal, 190 °C for 12 h	4.2 nm	NR	[52]
Goji Berry (Lycii Fructus)	CDs	Hydrothermal, 200 °C for 5 h	3.3 nm	17.2%.	[61]
Jackfruit (*Artocarpus heterophyllus*) peel and tamarind (*Tamarindus indica*) peel	N-CDs	Hydrothermal, 180 °C for 12 h	6.4 nm and 5.3 nm	QY of Jackfruit peel was 13.04% while that of tamarind peel was 6.13%.	[10]
Jatropha (*Jatropha curcas*) fruits	CDs	Carbonization, 5 h at 180 °C	3.2 nm	13.7%	[62]
Kiwifruit (*Actinidia deliciosa*) fruit	N-CDs	180 °C for 12 h	3.59 nm	NR	[63]
Kumquat (*Citrus Japonica*)	CDs	Microwave-assisted, at 630 W for 2 min	NR	7%	[64]
Lemon (*Citrus limon*) juice and onion (*Allium cepa*) juice	CDs	Microwave-assisted, power= 1450 W) for 6 min	6.15 nm	23.6%	[65]
Lemon (*Citrus limon*) peel	CDs	200 °C for 6 h	9.5 nm	11%.	[66]
Lemon (*Citrus limon*) peel waste	CDs	Hydrothermal 200 °C for 12 h.	1–3 nm	14%.	[67]
Mango (*Mangifera indica*) peels	CDs	Carbonization and oxygenolysis at 300 °C for 2 h to 6 h	2–6 nm	8.5%	[68]
Muskmelon (*Cucumis melo*) fruit	CMCDs	Acid oxidation at 80 °C for 15–30 min.	The B-, G-, and Y-CMCD were approximately 3.5, 4.3, and 5.8 nm, respectively.	B-, G-, and Y-CMCD were 14.3%, 26.9%, and 7.07%	[69]
Orange (*Citrus sinensis*) and limon (*Citrus limon*) peels	CDs	Heated at 180 °C for 2 h	0.35 and 0.37 nm	*Orange* and limon CDs were found to have QYs of 16.8% and 15.5%, respectively.	[48]
Orange (*Citrus sinensis)* peel and banana (*Musa* spp.) peel	CDs	Microwave for 2 × 5 min	NR	NR	[70]
Orange (*Citrus sinensis*) peels	CDs	Hydrothermal carbonization at 180 °C for 12 h	2–7 nm	36%.	[71]
Onion (*Allium cepa*) peels	CDs	Microwave treatment 1000 W at specific time intervals	NR	NR	[72]
Peanut (*Arachis hypogaea*) shells	CDs	Pyrolysis at 400 °C for 4 h in a laboratory furnace.	3.3 nm	10.58%	[73]
Pear (*Pyrus communis*) fruit	CDs	Hydrothermal 180 °C for 6 h	2.0 nm	NR	[48]
Papaya (*Carica papaya*) pulp waste	CDs	Pyrolysis	7 nm	23.7%	[74]
Pineapple (*Ananas comosus*) fruit	CDs	Acid oxidation, 80–100 °C, 15–60 min	B-, G-, and Y- CDs were 2.08, 2.8, and 4.0 nm, respectively.	B-, G-, and Y- CDs QY were 18.0%, 37.6%, and 44.7%, respectively.	[42]
Quince (*Cydonia oblonga*) fruit powder	CDs	Microwave irradiation, 220 °C in 1 min using 850 W. Hydrothermal, 200 °C for 4 h in furnace	4.85 nm	8.55%	[75]
Roasted chickpeas (*Cicer arietinum*).	CDs	Microwave-Assisted, 350 watts for 2 min	4.5–10.3 nm	1.8%.	[76]
Sago (*Metroxylon sagu*) waste	CDs	Pyrolysis temperature ranging from 250 °C to 450 °C for 1 h	6–17 nm	NR	[77]
Sapodilla (*Manilkara zapota*) fruits	CDs	Sonicated and heated at 100 °C for 60 min, 80 °C for 30 min, and 80 °C for 15 min	Blue, green, and yellow C-dots were 1.9, 2.9 and 4.5 nm, respectively	The QYs for the C-dots in blue, green, and yellow were 5.2%, 7.9%, and 5.7%, respectively.	[49]
Sugarcane bagasse (*Saccharum officinarum*), garlic (*Allium sativum*) peels, and taro (*Colocasia esculenta*) peels	CDs	Ultrasonic-assisted wet-chemical-oxidation method (~40 kHz, output power ~ 700 W)	8–12 nm	QY ranging from 4 to 27%.	[78]
Sweet Potato (*Ipomoea batatas*) peels	CDs	Hydrothermal, 200 C for 3 h.	2.0 nm	8.9%	[79]
Tomato (*Solanum lycopersicum*) fruits	CDs	Chemical oxidation method, 40 N H_3_PO_4_, and heated at 80 °C for 25 min.	5.0 to 10.0 nm	QY of blue, green, and yellow CDs were found to be 12.70%, 4.21%, and 2.76%, respectively.	[80]
Unripe Peach (*Prunus persica*)	N-CDs	Hydrothermal 180 °C for 5 h	8 nm	15%.	[81]
Vegetable waste	CDs	Treated for 45 min with ultrasound irradiation at 60 °C	6.03 nm	NR	[82]
Watermelon (*Citrullus lanatus*) peel	CDs	Carbonization, 220 °C for 2 h	2.0 nm	NR	[83]

Note: CDs—carbon dots; B—blue; Y—yellow; G—green; QY—quantum yield; NR—Not Reported.

**Table 2:** Antioxidant of carbon dots using agricultural extracts as carbon sources.

Plant Extract	Carbon Dots	Production Techniques	Key Findings	Reference
Beetroot (*Beta vulgaris*)	b-CDs	160 °C for 8 h, hydrothermal	The DPPH assay is used to assess the antioxidant properties of b-CDs. It yields a maximum scavenging activity of 94.5% at a dose of 1000 μg mL^−1^.	[148]
Black soya (*Glycine max*) beans	N-CDs	Pyrolyzed at 200 °C for 4 h	According to estimates, N-CDs had a final scavenging activity of 62.8% against DPPH. The maximum amount of superoxide anion that N-CDs could scavenge was 81.3%. DPPH and superoxide anion radicals were scavenged with 93.8% and 99.3% efficiency, respectively, using 1 μg·mL^−1^ ascorbic acid as a positive control.	[149]
Cumin (*Cuminum cyminum*) seeds	CDs	6 h at a temperature of 250 °C, hydrothermal	The concentration of CDs increased from 220 to 1540 μg/mL. The antioxidant capability of CDs increased by up to 80%, and the EC50 value was 1.2 mg/mL.	[150]
Dragon (*Hylocereus undatus*) fruit peels	CDs	Solvothermal treatment (acetic acid).	The antioxidant capacity of ACDs was very high; their DPPH radical scavenging IC_50_ value (0.70 μgmL^−1^) was significantly lower than that of the known antioxidant agent, ascorbic acid (4.34 μgmL^−1^).	[151]
Green (*Xinyang Maojian*) tea	CDs	200 °C for 3 h, hydrothermal	The linear regression result showed a good linear association between the inhibition value and the concentration of carbon dots when the concentration was between 1.40 and 11.20 μg·mL^−1^.	[152]
Lemons (*Citrus limon*) and onions (*Allium cepa*)	CDs	Hydrothermal, 200 °C for 3 h	At 100 μg/mL, LCDs, and OCDs displayed 80 and 90% radical scavenging activity at 100 μgmL^−1^.	[126]
Orange (*Citrus sinensis*) fruit peel	CDs	Sand bath at 180 °C under magnetic stirring for 12 h	Ascorbic acid and CDs were found to have estimated EC_50_ μg mL^−1^ values of 0.80 ug mL^−1^ and 4.73829, respectively.	[153]
Pineapple (*Ananas comosus*) waste	CDs	6 h at 200 °C, hydrothermal	The scavenging potential of CDs was 23.3% at a concentration of 5 mg/mL, whereas ascorbic acid exhibited the highest radical scavenging activity at the same dose, around 33.9%. At a dosage of 5 mg/mL, CDs scavenged the superoxide radical in a dose-dependent manner, reaching up to 42.9%; however, standard ascorbic acid demonstrated higher scavenging capacity (73.4% at 5 mg/mL). At a concentration of 5 mg/mL, CDs have hydrogen peroxide and hydroxyl scavenging activity of up to 93.4% and 50.2%, respectively.	[154]
Potato (*Solanum tuberosum*) Peel	CDs	200 °C for 6 h, hydrothermal	ABTS and DPPH approaches demonstrated significant antioxidant activity from the CDs, contingent upon the CD concentration.	[147]
Red cabbage (*Brassica oleracea*)	rcCDs	220 °C for 36 h, hydrothermal	Strong antioxidant properties were demonstrated by the rcCDs, which scavenged 61, 56, and 91% of the DPPH, hydroxyl, and potassium permanganate radicals, respectively.	[155]
Tea (*Camellia sinensis*) waste Grape (*Vitis vinifera*) pomace	TCDs GCDs	Carbonization method, 200 °C for 6 h in an oven. Hydrothermal-assisted process, 180 °C for 4 h in an oven	The DPPH radical scavenging activity of TCDs and GCDs was 75% and 56%, respectively, at a concentration of 375 µg·mL^−1^ CDs. For TCDs and GCDs, the EC_50_ values were 50 μg·mL^−1^ and 175 μg·mL^−1^, respectively.	[143]
Tomato (*Solanum lycopersicum*)	TCDs GCDs	160 °C for 3 h, hydrothermal	Carbon dots from tomatoes (TCDs) exhibit robust inhibition even at the lowest concentration, while carbon dots based on glutathione (GCDs) require a concentration at least four times higher to get equivalent antioxidant strength. The concentration required to achieve 50% of DPPH inhibition, or EC50, is estimated to be less than 4 ppm for TCD (0.16 ppm·nmol^−1^) and approximately 14 ppm for GCD (0.56 ppm·nmol^−1^).	[156]
Tumeric (*Curcuma longa*)	CD S-CDs	200 °C for 6 h, hydrothermal	At 200 lg/mL of CD, both CD and S-CD demonstrated significant free radical scavenging activity, around 90% and 80% observed in the ABTS method, and roughly 70% and 60% in the DPPH radical scavenging activity assay.	[157]
Waste (*Camellia sinensis*) tea	CDs	150 °C for 6 h, hydrothermal	The hydroxyl and superoxide radicals had IC50 values of 80 and 24.2 μg/mL, respectively.	[146]

Notes: CDs—carbon dots; DPPH—2,2-diphenyl-1-picrylhydrazyl; ABTS—2,2-azino-bis (3-ethyl-benzothiazoline-6-sulfonic acid; IC_50_—50% inhibition concentration; average effective scavenger concentration (EC_50_); TCD—Tea carbon dots; GCD—grape carbon dots.

**Table 3:** Microbial application of carbon dots from plant extracts.

Plant Extract	Carbon Dots	Production Techniques	Key Findings	Reference
Apple (*Malus* spp.) juice	CDs	Hydrothermal at 150 °C for 12 h	For bioimaging of *Mycobacterium tuberculosis* and *Pseudomonas aeruginosa* cells.	[172]
Beetroot (*Beta vulgaris*)	CDs	(100, 150, 200, 250 and 300 C) for 10 h/hydrothermal	The synthesized CDs exhibited noteworthy antibacterial activity against *Bacillus subtilis* and *Escherichia* coli bacteria, with a higher inhibition zone.	[185]
Dried papaya (*Carica papaya*) flesh	CDs	Thermal at 200 ° for 5 h	The CDs have also been demonstrated to be an excellent probe for *Escherichia coli* O157: H7 fluorescence sensing, with a 9.5 × 104 cfu mL^−1^ detection limit.	[186]
Lemons (Citrus limon) and onions (*Allium cepa*)	CDs	Hydrothermal 200 °C for 6 h	Agar-well diffusion method was used to evaluate he antifungal activity of both CDs screened against *Aspergillus* sp., *Candida albicans*, *Rhizopus* sp., *Botrytis cinerea*, and *Penicillium* sp. Both LCDs (lemon) and OCDs (onion) displayed inhibition zones ranging from 12.6 to 44.5 mm, respectively.	[126]
Papaya (*Carica papaya*) juice	CDs	Single-step hydrothermal at 170 °C for 12 h	When activated at 488 (green) and 561 (red) nm, CD-labeled *Bacillus subtilis* cells produced a strong green and red fluorescence, demonstrating that the cells efficiently absorbed the manufactured CDs. Similarly, when excited at 488 and 561 nm, CD-labeled *Aspergillus aculeatus* produced green and red fluorescence images.	[170]
Pear (*Pyrus pyrifolia*) fruit	CDs	Hydrothermal 180 °C for 6 h	Demonstrate that CDs can bioimage *Bacillus subtilis* bacterial cells, indicating the possibility of using the nanoprobe for cell imaging applications.	[49]
Pineapple (*Ananas comosus*) waste peels	CDs	200 °C for 6 h.	Antimicrobial against *Pseudomonas aeruginosa*, *Bacillus cereus* (28 mm), *Staphylococcus aureus* (25 mm), *Escherichia coli* (30 mm), and *Vibrio cholerae* (14 mm).	[127]
Potato (*Solanum tuberosum*) peel	CDs	200 °C for 6 h	According to the findings of the disk-diffusion and well-diffusion tests, the CDs demonstrated considerable effectiveness against *Listeria monocytogenes* but no inhibitory zone against *Escherichia coli*. The growth inhibition zones in the well-diffusion and disk-diffusion approaches were 7 mm and approximately 6 mm, respectively.	[147]
Sapodilla (*Manilkara zapota*)	CDs	Hydrothermal 80–100° for 15–60 min	The blue, green, and yellow C-Dots demonstrated promise as bioimaging agents for imaging the cells of *Aspergillus aculeatus*, *Escherichia coli*, and *Fomitopsis* sp.	[49]
Soya (*Glycine max*) chunks	CDs	Hydrothermal, 180 °C for 12 h	The investigated pathogens were not inhibited by ZnO/CDs nanocomposite, while ZnO NPs demonstrated a minimum inhibitory concentration screened against *Staphylococcus aureus* (19.53 μg/mL). The inhibitory impact of ZnO NPs is significantly diminished when CDs are present in ZnO/CDs nanocomposite.	[14]
Turmeric (*Curcuma longa*) extract	CDs	180 C for 10 h, solvent method	The antibacterial properties of CDs against *Escherichia coli* and *Staphylococcus aureus* under blue light irradiation were found to be dependent on carbonization levels, concentration, and light duration in vitro.	[187]
Turmeric (*Curcuma longa*)	CDs	200 °C for 6 h	The CDs demonstrated no antibacterial efficacy against *Escherichia coli* but showed strong antibacterial activity against *Listeria monocytogenes*.	[157]
Turmeric (*Curcuma longa*), lemon (*Citrus limon*), citric acid and grapefruit (*Citrus × paradisi*)	CDs	180 °C for 6 h	*Escherichia coli* pathogens were detected using CDs, a non-toxic photoluminescent sensor. The photoluminescence of the CDs nanocomposite was inhibited by increasing the number of *Escherichia coli* bacteria.	[188]

Notes: CD—carbon dots; ECN—EDA-doped carbon-based nanoparticles; ZnO—zinc oxide; CFU—colony-forming units.

**Table 4:** Biological studies of carbon-based nanoparticles for food packaging materials.

Polymer Matrix	Type of Carbon-Based NPs	Production Techniques and Condition	Key Findings	Reference
Carboxymethyl cellulose	CDs	Hydrothermally at 180 °C for 12 h	The polymer matrix contained uniformly distributed CDs, producing a highly translucent UV-blocking film. Increased the tensile strength by up to 27.6% and elastic modulus by up to 61.5%. Displayed excellent antioxidant and strong antimicrobial activity.	[23].
Gelatin/chitosan	CDs	Hydrothermal, 180 °C for 800 min	When compared to the gelatin/chitosan film, the gelatin/chitosan/CDs film exhibited improved UV shielding, antioxidant, and antibacterial properties with an optimal addition of 20% CDs.	[220]
Chitosan	N-CDs	Hydrothermal, 180 °C for 8 h	The CS/N-CDs composite film exhibits superior UV light barrier performance and strength when compared to the CS film. Improved mechanical properties and displayed high photodynamic antibacterial rates of 91.2% and 99.9% for *Escherichia coli* and *Staphylococcus aureus*, respectively, were demonstrated by the produced CS/7% N-CDs composites.	[124]
Chitosan	CDs	Hydrothermal, 180 °C for 12 h	Chitosan films incorporated with CDs demonstrated better mechanical, UV, and hydrophobic qualities. Reduction in populations of *Staphylococcus aureus* and *Escherichia coli* of about 3.19 and 2.05 Log10 CFU/mL, respectively, within 40 min.	[207]
Chitin nanowhisker (CNW) embedded soy protein	AgNP anchored carbon dots	Hydrothermal autoclave at 180 °C for 6 h	When CNW and AgNP were added, the mechanical strength and thermal stability of the SPI film were greatly enhanced, and the moisture content decreased. Superior antioxidant, antibacterial, and antifungal activities were conferred by the synergistic effect of AgNP and CNW.	[221]
Starch + anthocyanin	CDs	Hydrothermal, 180 °C for 5 h	CDs and *clitoria ternatea* flower (CTE) were distributed uniformly in the starch matrix, according to SEM, FTIR, and XRD analyses. Because of the complementary effects of CD and CTE films have the best mechanical, barrier, thermal, and antioxidant qualities.	[222]
Carboxymethylcellulose and agar-based	Nitrogen-doped polyethylene glycol-derived CD (NPCD)	Hydrothermal, 180 °C for 6 h	The NPCD-loaded film demonstrated strong antibacterial activity and high antioxidant levels (DPPH 12.7% and ABTS 67%). CMC/agar films incorporated with 8% NPCD prevented the proliferation of *Listeria monocytogenes* and *Escherichia coli*.	[205]
Gelatin/Carrageenan-Based	mushroom-CDs (mCDs)	Hydrothermal, 200 °C for 6 h	The addition of mCDs in the film significantly improved the mechanical characteristics, while its water vapor permeability and hydrophobicity remained mostly unchanged, and the films were highly transparent. Gelatin/carrageenan films with mCDs added showed significant antioxidant activity as assessed by DPPH and ABTS assays.	[223]
Cellulose nanofiber-based	Modified carbon dots with resazurin (R-CD)	Hydrothermal, 160 °C for 6 h	The CNF/R-CD indicator film exhibited enhanced thermal stability and a marginally reduced water contact angle in comparison to the clean CNF film. UV-barrier qualities of the CNF/R-CD film were good, as evidenced by 98.3% and 87.7% of UV-B and UV-A light barriers, respectively.	[203]
Nanocellulose oxidized by applying TEMPO oxidation	CDs and ZnO	One-step microwave reaction at 600 W and heated at 200 °C for 2 min	Films demonstrated better UV-blocking capabilities, superior thermal stability, and excellent transparency to visible light. When the same amounts of ZnO were used in CDs-ONC-ZnO films, the UV-blocking ratio (UVR) was significantly higher than in previously proposed NC-ZnO films. Furthermore, CDs-ONC-s-ZnO film with 4 wt% sheet-like ZnO (s-ZnO) at 300 and 225 nm has a higher UVR (92.74% and 98.99%) than CDs-ONC-b-ZnO film supplemented with belt-like ZnO (b-ZnO) and CDs-ONC-p-ZnO film under the same conditions.	[224]
Chitosan nanocomposite hydrogel films	CH-CDs	Hydrothermal, 200 °C for 8 h	Chitosan + CDs hydrogel films were found to have better UV-visible blocking. Transmittance for CH-CD4 was up to 20% lower than that of CH hydrogel film in the 300–600 nm wavelength range. Compared to CH hydrogel film (5.1 MPa), the tensile strength (TS) of the CH-CD1 nanocomposite film increased significantly to 18.6 MPa. The hydrophobicity of the hydrogel nanocomposite films was indicated by an increase in contact angle values, which went from 64.95° for CH films to 88.75° for CH-CD3 films.	[24]
Nanocellulose	CDs	Hydrothermal, 160 °C for 6 h	*Listeria monocytogenes* was used to test antibacterial activity. C-dots considerably enhanced the tensile strength and reduced strain in relation to breaking BNC when added to BNC. C-dots were used to create a BNC sheet with highly effective UV-blocking properties.	[225]
Polyvinyl Alcohol	CD	Hydrothermal, 200 °C for 8 h	The composite’s tensile strength and modulus increase dramatically when CNF and CDs are added to the PVA matrix. PVA-based films become more water-resistant when CNF and CDs are doped. When the produced films contain more CDs, the water contact angle reduces, and their wettability improves.	[226]
Chitosan/gelatin-based	CDs	Hydrothermal, 200 °C for 6 h	The composite film exhibited a notable improvement in UV protection qualities but a slight drop in transparency. The produced film demonstrated a high level of antioxidant efficacy, with >74% DPPH and 100% ABTS radical scavenging capacity. Additionally, the Chitosan/gelatin-based films demonstrated strong antibacterial efficacy against *Listeria monocytogenes*.	[208]
Pectin/gelatin-based	CD	Hydrothermal, 200 °C for 6 h	The CD-added film demonstrated excellent UV protection qualities without significantly affecting the transparency of the pectin/gelatin film. Hydrophobicity, vapor permeability, and mechanical properties of the film were impacted by the addition of CD. Furthermore, the DPPH and ABTS tests revealed that the pectin/gelatin-based films with CD added had strong antioxidant activity. Also, the sulfur-functionalized CD film exhibited potent antibacterial activity against *Listeria monocytogenes* and *E. coli*.	[141]
Polyvinyl Alcohol	CDs	Hydrothermal, 200 °C for 8 h	Emission intensity of the PVA/CNF/CDs films decreased gradually. Increased water absorption rate. The light barrier of the composite improved.	[227]
Nanocellulose film	CDs	Hydrothermal, 200 °C for 2 h	Nanocellulose with CDs improved flexibility in comparison to neat nanocellulose. Improved ultraviolet barrier property and prevented the growth of Gram-positive bacteria more than Gram-negative bacteria.	[141]
Polyvinyl Alcohol	CDs	Hydrothermal, 200 °C for 8 h	Emission intensity of the PVA/CNF/CDs films decreased gradually. Increased water absorption rate. The light barrier of the composite improved.	[227]
Polyvinyl alcohol (PVA)	CDs	Hydrothermal, 200 °C for 5 h	Improved UV barrier properties of the composite PVA films. PVA@WTR-CDs-3 films were able to completely block (100%) the UV-C (230–280 nm) and UV-B (280–315 nm) regions, and only 20–60% of the UV-A (315–400 nm) region. PVA@WTR-CDs-5 composite films also achieved a maximum UV barrier. No significant changes in the intrinsic mechanical and tensile strength of PVA films. No changes were observed in the thermal analysis study when WTR-CDs were incorporated in PVA films.	[228]
Chitosan and PVA	CDs	Hydrothermal, 200 °C for 8 h	The PVA/CS/1-CDs film demonstrated a UV-A barrier capacity of 83.58% and a transparency of 72.34%, respectively. While PVA/CS/2-CDs film achieved a UV-A barrier capacity of 94.53%.	[229]
Chitosan-polyvinylpyrrolidone	N-doped carbon dots (NCDs)	Hydrothermal, 200 °C for 12 h	The film with NCDs integrated displayed a smooth surface with evenly dispersed NCDs in the chitosan-PVP film. while NCDs with chitosan-PVP-orange peel film had a uniformly smooth surface. Chitosan-PVP films with NCDs displayed better tensile strength, elongation at the break, reduced moisture content, a contact angle value of 89.6°, and showed degradation exceeding 40% over a 50-day period.	[230]
Cellulose nanofiber-based	Glucose (GCD) and N-functionalized CDs (NGCD)	Hydrothermal, 200 °C for 6 h	GCD and NGCD reduced T280 by 91–28% and T660 by 12–10% while maintaining high UV blocking qualities for the CNF film without affecting its transparency. No changes in mechanical properties were observed when GCD and NGCD were added, but the films’ water vapor permeability (WVP) and water contact angle (WCA) increased. The composite films incorporated with GCD and NGCD exhibited a high level of antioxidant activity, scavenging 80–85% of DPPH radicals and 99% of ABTS. The CNF/NGCD film demonstrated better antibacterial and antifungal activity than the CNF/GCD film.	[125]
Chitosan	Nitrogen and phosphorus (NP-CDs)	Hydrothermal, 180 °C for 8 h	The NP-CDs increased the density of the film, water contact angle from 79.2° to 105.8°, UV-A and UV-B transmittance, and antibacterial activity to both E. coli and S. aureus compared to the chitosan film. However, NP-CDs reduced water vapor permeation.	[231]
Agar-based film	Ag-CMCDs	Hydrothermal, 180 °C for 12 h	Agar-based films had high UV barrier properties as well as good biodegradability, with roughly 86% degradation in 60 days. Improved tensile strength value to 41.85 MPa. In addition, the agar-based films demonstrate excellent antibacterial activity against *Staphylococcus aureus* and *Escherichia coli*.	[232]
Gelatin-based	CDs	Hydrothermal, 200 °C for 6 h	When CDs were added, the water vapor permeability (by 28%) and hydrophobicity (by 9% and 13%) of the very transparent film significantly improved without affecting the mechanical properties. The gelatin films with CD added showed strong antioxidant activity and UV barrier properties. Moreover, gelatin-based films with CDs significantly improved antibacterial efficacy.	[147]
Carboxymethyl cellulose	CDs	Hydrothermal, 200 °C for 5 h	Improved structural homogeneity, optical properties, and tensile strength.	[233]

Notes. CD: carbon dots, N-CDs: nitrogen-doped carbon dots; NP-CDs: nitrogen and phosphorus carbon dots; Ag: silver nanoparticle; CMC: carboxymethyl cellulose, G: gelatin; PVA: polyvinyl alcohol; CNF: cellulose nanofibers; CH: chitosan; WCA: water contact angle; WVP: water vapor permeability; UV: ultraviolet; TS: tensile strength; CFU: colony-forming units; CTE: *clitoria ternatea* flower; WTR: waste tea residue.

**Table 5:** Formulation and application of carbon dot coatings on food products.

Food Product	Polymer Matrix	(%) Weight of CDs	Storage Condition	Impact of Coating	Reference
Avocado (*Persea americana*)	Chitosan + gelatin	CDs 1% and 2% wt	25 °C for 21 days	On day 14, mold growth was noted. The antifungal activity of CDs films was dependent on CDs concentration.	[184]
Banana (*Musa* spp.)	Polyvinyl alcohol (PVA)	CDs at 0.5%	23 °C	The appearance of bananas coated with CS-CDs/PVA showed less decay compared to those coated with PVA and the control (no coating). The bananas gradually deteriorated during storage, as seen by black spots.	[55]
Lemon (*Citrus limon*)	Carboxymethyl cellulose	CDs 3.0 wt%	Room humidity at 25 °C for 21 days	Retained their original flavor and color, and the surface of the lemons displayed no signs of mold growth.	[23]
Sliced tomatoes (*Solanum lycopersicum*)	Agar based	CD and AgNO_3_ 1, 2, and 3%	Ambient temperature with 50% RH for 5 days	Reduced microbial growth, minimal water loss, and delayed the decay of tomatoes and prolonged shelf life.	[232]
Fresh-cut cucumber (*Cucumis sativus*)	Chitosan	CDs at 0%, 1.5%, 3%, and 4.5%	4 ∘C for 15 days.	During storage, the total number of colonies, mold, and yeast growth in fresh-cut cucumbers packaged in a regulated environment was suppressed by the CDs/CH coating. Moreover, the 4.5% CDs/CH coating successfully inhibited peroxidase activity, lowered water mobility, and prevented weight, firmness, and total soluble solids losses in fresh-cut cucumbers during storage. It also prevented the ascorbic acid content and flavor from degrading.	[243]
Fresh-Cut Cucumber (*Cucumis sativus*)	Chitosan and ultrasound	CDs at 4.5%	4 °C for 15 days	The findings showed that after 15 days of storage, US treatment coupled with CDs coating significantly reduced the overall bacterial count to 5.18 log CFU g^−1^, mold, and yeast to 3.45 log CFU g^−1^. The treatment also obtained a lower weight loss of 8.54%, respiration rate of 4.67 mg kg^−1^ h^−1^ CO_2_ and malondialdehyde content of 2.24 μmol kg^−1^. Furthermore, the US treatment inhibited polyphenol oxidase activity to 137.17 U kg^−1^ s ^−1^ and peroxidase activity to 139.83 U kg^−1^ s^−1^. They also maintained higher firmness of 6.78 N, ascorbic acid content of 0.0243 g kg^−1^ and total soluble solids of 2.29 °Brix, and after 15 days of storage, there was less of a change in the water content.	[142]
Fresh-cut pears (*Pyrus communis*)	Carboxymethyl chitosan	CDs at 1, 2 and 3%	4 °C for 5 days	The CS/2%PER-CDs coating solution effectively reduced respiration (51.67 mgCO2/Kg⋅h) and ethylene production rate (0.75 μg/kg⋅h).	[245]
Litchi fruit (*Litchi chinensis*)	Silk sericin and chitosan	CDs at 20%	25 °C and 85% relative humidity for 6 days	Effectively reduced water loss, nutritional components, such as ascorbic acid and soluble solids	[248]
Litchi fruit (*Litchi chinensis*)	Chitosan (CS)	CDs	25 °C and 85–90% RH	Litchi fruit treated with EA/CS/CDs displayed higher levels (L*, a*, and b*) compared to the control treatment. Maintain higher levels of vitamin C, sucrose, and total sugar to ensure optimal nutritional quality and minimize weight loss. Effectively postponed the pericarp from browning.	[249]
Strawberries (*Fragaria × ananassa*)	Gelatin-based	R-CDs	Room temperature for 8 days	Most of the strawberries in the control group were shriveled or bacterially contaminated by the end of the eighth day of storage, with a rotting rate of almost 75%. In contrast, most of the strawberries coated with Gelatin + 1.5% R-CDs had a rotting rate of roughly 4.17%. On the fourth day, the control group showed a significant weight reduction, yet the coated group that contained 1.5% R-CDs experienced comparatively less weight loss. This pattern persisted until the last day of storage, when the 1.5% R-CDs group experienced a weight loss rate of 29.63%, and the control treatment was 45.47%.	[235]
Strawberries (*Fragaria × ananassa*)	Packaging material	CDs	25–27 °C and 68% RH	Weight loss on the fourth day of observation was 5.21% for strawberries packed with OCDs and 8.14% for those packaged with LCDs, compared to 18.71% for the control. The rate of decay for OCDs was 11%, LCDs was 40%, and control was 96.65% on the same day, as shown in Figure 12.	[126]
Tangerine (*Citrus reticulata*) and strawberry (*Fragaria × ananassa*) fruit	Cellulose nanofiber-based coating	GCD and NGCD at 1 wt % based on polymer	NR	Mold developed on the surface of the uncoated and coated fruit with the neat CNF and CNF/GCD films; the mold became worse after 15 and 4 days of storage. CNF/NGCD film maintained better quality without exhibiting any signs of mold growth.	[125]
Tomato (*Solanum lycopersicum*)	Pure gelatin cellulose nanofiber (CNF)	CDs	28 °C with 40% humidity	Compared to CK, the hardness reduction in the G/10CD/3CNF/EON treatment condition was slower, demonstrating that the intricate layer that formed a barrier around the tomato helped preserve the fruit firmness by preventing water loss. Moreover, G/10CD/3CNF/EON treatment maintained soluble solids content and TA better than EON and the control group.	[122]

Notes. CD: carbon dots; N-CDs: nitrogen-doped carbon dots; CMC: carboxymethyl cellulose; PVA: polyvinyl alcohol; G: gelatin; CS: chitosan; WVP: water vapor permeability; CNF: cellulose nanofibers (CNFs); PLA: polylactic acid; CFU: colony-forming units; L* (lightness); a* (red/green); b* (yellow/blue).

## Data Availability

Data are contained within the article.

## Abbreviations

The following abbreviations are used in this manuscript:

CDs	Carbon dots
CNT	Carbon nanotubes
ABTS	2,2′ -azino-bis (3-ethyl-benzothiazoline-6-sulfonic acid
DPPH	2,2-diphenyl-1-picrylhydrazyl
IC_50_	50% inhibition concentration
AMR	Antimicrobial resistance
CAGR	Compound Annual Growth Rate
CNF	Cellulose nanofibers
CMC	Carboxymethyl cellulose
GO	Graphene oxide
HDPE	High-density polyethylene
FTIR	Fourier Transform Infrared spectroscopy
LDPE	Low-density polyethylene
PCL	Polycaprolactone
PVC	Polyvinyl chloride
PET	Polyethylene terephthalate
PLA	Poly lactic acid
PBS	Polybutylene succinate
QY	Quantum yield
UV	Ultraviolet
AFM	Atomic force microscopy
SEM	Scanning Electron Microscopy
ROS	Reactive oxygen species
SET	Single electron transfer
TS	Tensile strength
EVOH	Thylene vinyl alcohol copolymer
TEM	Transmission Electron Microscopy
TGA	Thermogravimetric analysis
PL	Photoluminescence
WVP	Water vapor permeability
WHO	World Health Organization
XRD	X-ray Diffraction
XPS	X-ray Photoelectron Spectroscopy
ZnO	Zinc oxide
